# Unlocking Superior Stability in High-Salinity Oxygen Evolution Reaction: A Ru Stabilized NiFeOOH/Ni Anode with over 2000 h Durability

**DOI:** 10.1007/s40820-026-02072-4

**Published:** 2026-01-26

**Authors:** Jin He, Haoyun Sheng, Yichao Lin, Bingqi Gong, Yayun Zhao, Ziqi Tian, Liang Chen

**Affiliations:** 1 Zhejiang Key Laboratory of Advanced Fuel Cells and Electrolyzers Technology, Ningbo Institute of Materials Technology and Engineering, Chinese Academy of Sciences, Ningbo, 315201 Zhejiang People’s Republic of China; 2 School of Materials Science and Engineering, Zhejiang University of Technology, Hangzhou, 3100143 Zhejiang People’s Republic of China; 3 School of Materials Science & Chemical Engineering, Ningbo University, Ningbo, 315211 Zhejiang People’s Republic of China; 4 University of Chinese Academy of Sciences, Beijing, 100049 People’s Republic of China

**Keywords:** Saline water electrolysis, Oxygen evolution reaction, Chloride corrosion, Electrostatic repulsion, NiFeOOH

## Abstract

Saline water electrolysis presents a promising pathway for green hydrogen production by leveraging abundant saline water resources instead of scarce freshwater. However, the presence of highly corrosive chloride ions (Cl^−^) severely undermines anode durability. This instability arises from two main issues: (i) penetration of Cl^−^ through catalyst layers to the underlying substrate and (ii) degradation of active catalytic sites due to Cl^−^ attack. To tackle both issues simultaneously, we introduce ruthenium (Ru) ions as a dual-function stabilizing agent in NiFe-based anodes. Our results show that Ru incorporation promotes the formation of a protective surface layer enriched with Ru atoms, along with a denser NiFeOOH catalyst structure, which collectively inhibit Cl^–^ infiltration. Moreover, atomically dispersed ruthenium (Ru_SA_) within the NiFeOOH matrix effectively mitigates Cl^–^-induced corrosion of active sites. Thanks to this dual stabilization effect, the resulting Ru_SA_-NiFeOOH/Ni anode exhibits exceptional operational stability—over 2000 h at an industrial current density of 0.5 A cm^−2^ in a chloride-enriched alkaline medium (1 M KOH + 2 M NaCl)—setting a new benchmark for performance under such aggressive conditions. This study establishes a robust dual stabilization strategy that significantly enhances anode stability in saline water electrolysis.

**Supplementary Information:**

The online version contains supplementary material available at 10.1007/s40820-026-02072-4.

## Highlights

- A dual-function stabilizing agent in NiFe-based anodes is proposed.
- Ru incorporation promotes the formation of a protective surface layer enriched with Ru atoms, along with a denser NiFeOOH catalyst structure.
- Ru_SA_-NiFeOOH/Ni anode exhibits exceptional operational stability over 2000 h at an industrial current density of 0.5 A cm^−2^ in a chloride-enriched alkaline medium.

**Supplementary Information:**

The online version contains supplementary material available at 10.1007/s40820-026-02072-4.

## Graphical abstract

**Figure:**
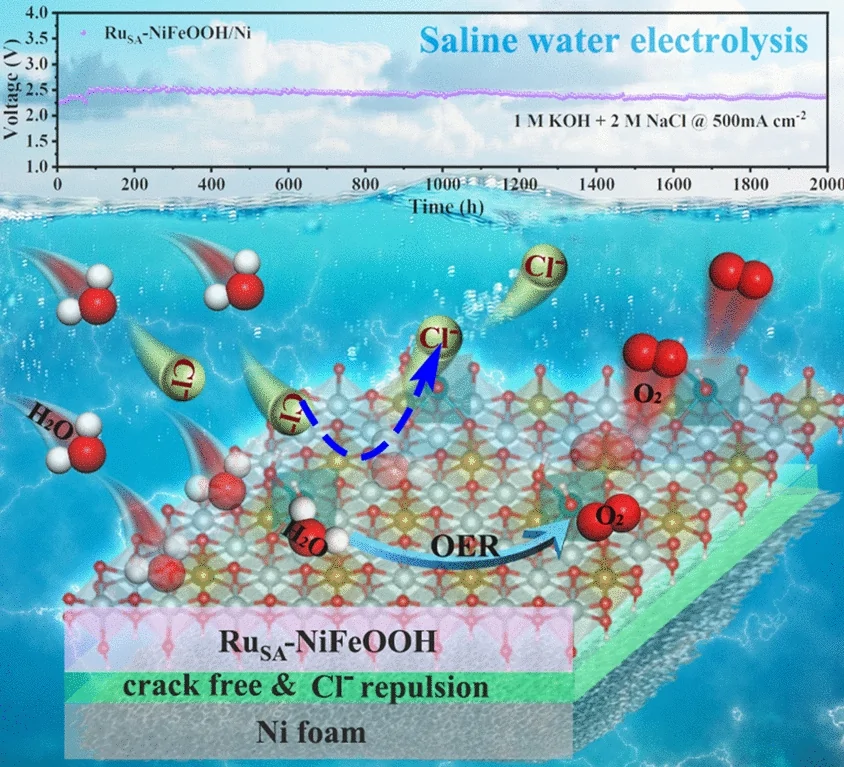

## Introduction

The relentless increase in global energy consumption, fueled by industrialization and technological advancement, has deepened reliance on fossil fuels [1, 2]. This dependence aggravates environmental degradation and climate change due to substantial greenhouse gas emissions [3, 4]. Hydrogen (H_2_) energy has emerged as a promising alternative, offering carbon-free utilization and efficient interconversion with electricity through electrolysis and fuel cells [5–7]. However, current hydrogen production remains largely carbon-intensive, with 96% originating from steam methane reforming and coal gasification—processes that emit 9–12 kg of CO_2_ per kilogram of hydrogen produced [8]. Water electrolysis, especially when coupled with renewable energy, represents a viable route to green hydrogen production [9–11]. However, the scarcity of freshwater resources has spurred interest in saline water electrolysis, which leverages Earth’s abundant saline water sources—such as seawater and salt lake water—as the electrolyte. Despite its potential, this method still faces major challenges, particularly anode instability in chloride-rich environments [12–14].

Significant efforts have been devoted to improving anode stability in saline water electrolysis through the development of anti-Cl^−^ corrosion catalyst layers, including transition metal sulfides [15, 16], nitrides [17, 18], and phosphides [19–21]. Notably, catalyst restructuring is a prominent phenomenon during water electrolysis, with these catalysts typically undergoing oxidation and restructuring into (oxy)hydroxides during the oxygen evolution reaction (OER) [22]. Thus, corrosion resistance cannot be simply attributed to the original structure, which significantly increases the challenge of fully elucidating the fundamental mechanisms underlying chloride resistance. For example, our previous study showed that NiFeP undergoes surface reconstruction to form NiOOH@FeOOH under alkaline saline conditions [23]. On the other hand, existing studies often attribute anode degradation to the corrosion of active sites within the catalyst layer by Cl^−^, leading to metal leaching and performance decay. This view appears plausible given that NiFe-layered double hydroxide grown on nickel foam (NiFe-LDH/Ni)—a state-of-the-art OER catalyst—exhibits limited stability in saline electrolytes. However, from the perspective of corrosion chemistry, it is unlikely that well-formed metal (oxy)hydroxides are readily corroded by Cl^−^. In a recent study, we demonstrated that the instability of NiFe-LDH/Ni arises primarily from cracks in the catalyst layer, which allow Cl^−^ penetration to the underlying metallic Ni substrate, resulting in severe corrosion of the substrate rather than the catalyst itself [24]. Therefore, instead of focusing solely on developing stable OER catalysts, greater attention should be directed toward protecting the inner metallic substrate of the anode.

Ruthenium oxide has served as a dimensionally stable anode in the chlor-alkali industry for over six decades [25]. Ru sites are known to exhibit exceptional stability in Cl^−^-rich electrolytes and demonstrate a high affinity for Cl^−^ ions. Meanwhile, recent advances have emphasized electronic structure modulation of isolated metal sites and interface engineering as the key strategies for enhancing electrocatalytic performance [26–28]. In this work, we demonstrate that incorporating Ru into NiFeOOH dually stabilizes the NiFeOOH/Ni electrode: (i) by promoting the formation of a Ru concentrated protective layer on the Ni substrate and (ii) through atomic dispersion of Ru (Ru_SA_) within the NiFeOOH matrix to protect catalytic sites from Cl^–^ attack. The resulting Ru_SA_-NiFeOOH/Ni electrode achieves an ultralow overpotential of 220 mV at 100 mA cm^−2^ in simulated saline water (1 M KOH + 0.5 M NaCl). Notably, it exhibits exceptional operational stability—maintaining performance for over 2000 h at 0.5 A cm^−2^ in Cl^–^-enriched alkaline medium (1 M KOH + 2 M NaCl)—whereas state-of-the-art NiFe-LDH/Ni and NiFeOOH/Ni anodes fail within 15 h under the same conditions. In situ vibrational spectroscopy and electrochemical analysis reveal that Ru promotes the irreversible oxidation of Ni^2+^ to Ni^3+^, leading to the formation of a robust and compact catalyst layer that effectively blocks Cl^−^ penetration toward the substrate. Furthermore, we show that atomically dispersed Ru creates a localized Cl^−^-enriched region around Ru sites, which electrostatically repels Cl^−^ ions and thereby shields adjacent catalytic sites (Ni/Fe) from corrosion.

## Experimental Section

### Preparation of Ru_SA_-NiFeOOH/Ni

In situ growth of Ru_SA_-NiFeOOH/Ni on Ni foam was carried out using a dipping method. Prior to the growth of Ru_SA_-NiFeOOH/Ni, Ni foams (2 × 3 cm^2^) were sequentially sonicated in acetone, hydrochloric acid solution (1 M), deionized water, and ethanol for 15 min, then Ni foams were preserved in ethanol for subsequent use. The precursor solution was prepared by adding 4.04 g of Fe(NO_3_)_3_·9H_2_O and 50 mg of RuCl_3_ to 50 mL of deionized water and dissolved under stirring. Ni foams stored in ethanol were dried using mirror paper, followed by immersion in the precursor solution for 1 min, during which they were turned over at 30 s intervals. After immersion, the obtained Ru_SA_-NiFeOOH/Ni was dried in a vacuum oven at 60 °C overnight. To evaluate the effect of Ru loading, a series of samples were prepared through adding different amounts of RuCl_3_ (25, 50, 100, 150, and 200 mg). The preparation for the control samples (NiFeOOH/Ni and NiFe-LDH/Ni) is provided in the Supporting Information.

### Characterizations

The crystal structure of the samples was analyzed using X-ray diffractometer (XRD, Bruker D8 Advance Davinci, German) with Cu-Kα radiation (*λ* = 1.54178 Å). XRD spectra were acquired over the range of 5°–80°. The analysis and background correction of XRD patterns were implemented using Jade software. The morphology and microstructure of the materials were investigated through scanning electron microscopy (SEM) using a Hitachi S-4800 cold field emission scanning electron microscope. Transmission electron microscopy (TEM) measurements were performed using a JEOL JEM-F200 microscope. Additionally, high-angle annular dark-field scanning transmission electron microscopy (HAADF-STEM), high resolution transmission electron microscopy (HRTEM), selective electron diffraction (SAED), and energy-dispersive X-ray spectroscopy (EDS) elemental maps were acquired. The sample used for cross-sectional EDS elemental maps was obtained on a double-beam scanning electron microscopy (FIB, Helios-G4-CX). All samples for TEM measurements were prepared by ultrasonic dispersion in ethanol and drop-cast onto copper grids coated with a carbon film. TEM images were processed with DigitalMicrograph software (Gatan). The aberration-corrected high-angle annular dark-field scanning TEM (AC-HAADF-STEM) with atomic resolution was used to distinguish the states of doped Ru species in the nanosheet matrix. X-ray photoelectron spectroscopy (XPS) was carried out using a Kratos AXIS Supra spectrometer. The XPS peak fitting and data analysis were carried out using CasaXPS software. The binding energy of all peaks was calibrated with respect to C 1*s* peak at 284.8 eV. Inductively coupled plasma optical emission spectroscopy (ICP-OES) analysis was performed using a SPECTRO ARCOS plasma emission spectrometer (Germany) to determine the Ru elemental content of the catalyst.

### Electrochemical Measurements

#### Polarization Measurements

The corrosion behaviors of the samples were investigated through potentiodynamic polarization measurements using a traditional three-electrode system on an electrochemical workstation, which consisted of the prepared samples as the working electrode (1 × 1 cm^2^), a Pt foil counter electrode (1 × 1 cm^2^), and a Hg/HgO reference electrode. Prior to the polarization measurements, the open circuit potential (OCP) was monitored until reaching a stable equilibrium state (typically several h). Potentiodynamic polarization curves were obtained by scanning the potential from − 0.4 to 0.4 V (vs. Hg/HgO) at a scan rate of 10 mV s^−1^. The corrosion potential (*E*_*corr*_) was calculated through Tafel extrapolation, defined as the electrode potential at which the cathodic reduction rate and the anodic oxidation rate reach dynamic equilibrium, resulting in a net current density of zero across the electrode interface.

#### Electrochemical In Situ Raman Measurements

Electrochemical in situ Raman spectroscopy was carried out using a RENISHAW Raman spectrometer equipped with a Leica TCS SP8 CARS microscope and a Spectra-Physics Ar laser (532 nm). All in situ Raman measurements were performed with a customized three-electrode spectroelectrochemical cell at room temperature, which comprised the as-synthesized material as the working electrode, a Pt wire counter electrode, and an Ag/AgCl (3.5 M KCl) reference electrode, immersed in 1 M KOH + 0.5 M NaCl electrolyte solution. Electrochemical control was maintained using chronoamperometry, with applied potentials ranging from 1.025 to 1.525 V versus. RHE. Prior to each Raman measurement, the working electrode was stabilized at the target potential for 40 s to ensure steady-state conditions, during which the potential was maintained constant.

#### Electrolyzer Assembly and Testing

Long-term stability tests were performed in a circulating flow-type electrolyzer using commercial Pt/Ni (cathode) and Ru_SA_-NiFeOOH/Ni (anode, geometric area: 2 × 2 cm^2^), separated by a polyphenylene sulfide (PPS) membrane. A total of 2 L electrolyte (6 M KOH saturated with NaCl) was continuously circulated between the electrolyzer and the reservoir at 100 mL min^−1^ using a peristaltic pump. Electrochemical performance and durability measurements were conducted at 55 °C using a LANHE battery tester, effectively suppressed the salt crystallization observed at room temperature and prevented flow-channel blockage. During long-term operation, deionized water was periodically replenished to compensate for water loss, thereby maintaining the electrolyte level at a fixed marked position and preventing concentration fluctuations.

## Results and Discussion

### Morphology and Structural Characterization

**Fig. 1:**
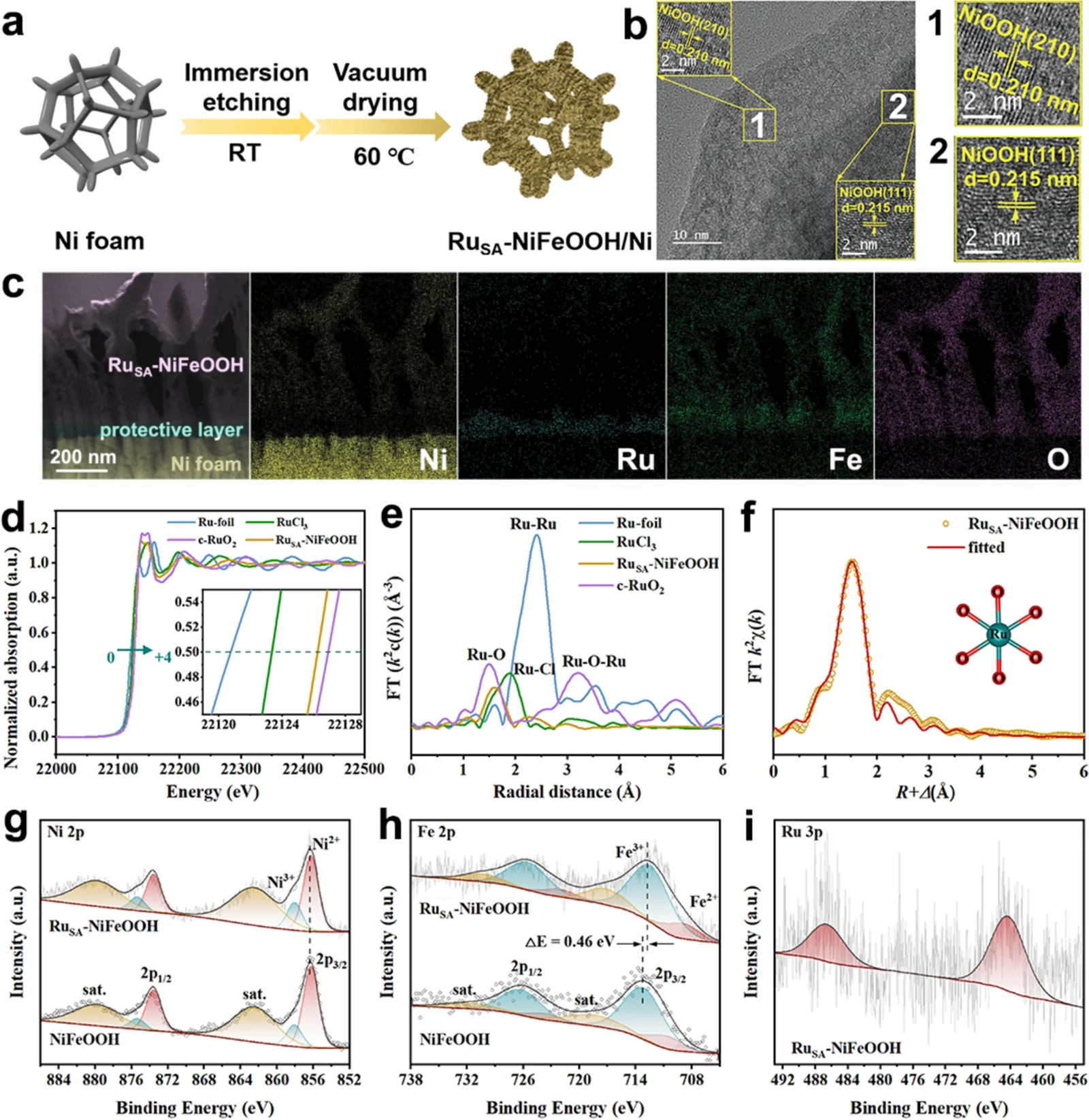
Synthesis illustration and structural characterization of Ru_SA_-NiFeOOH/Ni. **a** Synthesis diagram for Ru_SA_-NiFeOOH/Ni; **b** HRTEM images, and **c** cross-sectional elemental mapping images of Ru_SA_-NiFeOOH/Ni; **d** XANES spectra of Ru K-edge (the inset shows the enlarged absorption edge, demonstrating the Ru valance change), **e** *k*^2^-weighted Fourier transform spectra from EXAFS of Ru_SA_-NiFeOOH, RuO_2_, RuCl_3_ and Ru foil; **f** FT-EXAFS fitting spectrum at Ru K-edge and simulated atomic model of Ru_SA_-NiFeOOH; **g** Ni 2*p* XPS spectra, **h** Fe 2*p* XPS spectra, and **i** Ru 3*p* XPS spectra of NiFeOOH and Ru_SA_-NiFeOOH

The Ru_SA_-NiFeOOH/Ni was prepared by immersing Ni foam in a solution containing oxidative Fe^3+^ and Ru^3+^ ions, as illustrated in Fig. 1a. SEM image reveals that the Ru_SA_-NiFeOOH possesses nanosheet morphology, and the nanosheets are interconnectedly grows on Ni substrate, resulting a three-dimensional porous structure (Fig. S1). In contrast, when the RuCl_3_ precursor was absent in the impregnation, a three-dimensional bouquet-like structure consisting of nanorods clusters was formed on Ni substrate (NiFeOOH/Ni, Fig. S2). It suggests that the presence of Ru^3+^ promote the formation of NiFeOOH nanosheet, which may be attributed to the participation of Ru^3+^ in the NiFeOOH formation [29]. TEM characterizations further clearly demonstrate that the Ru_SA_-NiFeOOH exhibited a nanosheet architecture, while NiFeOOH exhibits a nanorod-dominated morphology (Figs. S3a and S4a). The as-prepared Ru_SA_-NiFeOOH or NiFeOOH exhibits very poor crystallinity, as revealed by HRTEM images and SAED analysis (Figs. 1b, S3b, and S4b, c). As shown in Fig. S5, the characteristic vibrational modes corresponding to FeOOH species are clearly observed in the Raman spectroscopy of Ru_SA_-NiFeOOH, indicating that Fe predominantly exists in the FeOOH configuration within the NiFeOOH matrix [30, 31]. The XRD patterns of Ru_SA_-NiFeOOH/Ni and NiFeOOH/Ni are shown in Fig. S6, which only exhibits the signals for metallic Ni substrate, which can be attributed to the poor crystallinity of the product as observed in the HRTEM image and/or the small amount of the product formed on the Ni surface. AC-HAADF-STEM imaging was employed to investigate the dispersion state of Ru within the Ru_SA_-NiFeOOH framework. As shown in Fig. S7, a limited number of isolated bright spots are visible at the atomic scale, which can be assigned to individual Ru atoms owing to the larger *Z*-contrast relative to Ni and Fe. Importantly, the bright spots are spatially well separated, and no Ru clusters or nanoparticles are detected, indicating an atomically dispersed Ru species. Cross-sectional EDS elemental mapping reveals that Ru is predominantly concentrated at the interface between the catalyst layer and the Ni foam substrate (Fig. 1c). This interfacial Ru-enriched region forms a protective layer that effectively suppresses Cl^–^-induced corrosion of the Ni foam during the saline water electrolysis. In contrast, only a limited amount of Ru is present within the Ru_SA_-NiFeOOH nanosheet matrix, consistent with the ultralow Ru loading and accounting for the absence of detectable Ru signals in planar EDS mapping. ICP analysis indicated that Ru accounts for 0.62 atom% of the total metal atoms in Ru_SA_-NiFeOOH (Table S1).

X-ray absorption near-edge spectroscopy (XANES) and extended X-ray absorption fine structure (EXAFS) analyses were employed to investigate the atomic structure of Ru_SA_-NiFeOOH, with Ru foil, RuCl_3_, and commercial RuO_2_ (c-RuO_2_) as references. As shown in Fig. 1d, the normalized Ru K-edge absorption edge position of Ru_SA_-NiFeOOH lies between those of RuCl_3_ and c- RuO_2_, indicating an intermediate oxidation state, which was quantified to be + 3.43 (Fig. S8). The Ru K-edge EXAFS spectra further reveal that Ru_SA_-NiFeOOH exhibits a single dominant Ru–O peak at 1.60 Å, with no detectable Ru–Ru, Ru–O–Ru or Ru–Cl scattering contributions, ruling out Ru clustering as well as residual RuCl_3_ species (Fig. 1e). This observation confirms the atomic dispersion of Ru within the matrix, in good agreement with the AC-HAADF-STEM observations. The slightly longer Ru–O bond length compared to c-RuO_2_ is attributed to support-induced distortion and weakened Ru–O covalency [32]. The absence of pronounced Ru–O–M (M = Ni/Fe) higher-shell features, together with surface-localized Ru atoms observed by AC-HAADF-STEM, suggests that Ru single atoms are anchored on the surface of NiFeOOH rather than substituting lattice sites [33]. EXAFS fitting yields a Ru–O coordination number (C.N.) in Ru_SA_-NiFeOOH is close to 6 (C.N. 5.1 ± 0.5), indicative of an octahedral-like oxygen environment (Fig. 1f and Table S2) [34].

XPS was further employed to examine the surface elemental composition and valence states of the as-prepared samples. As shown in Figs. 1g–i, the presence of Ru 3*p* XPS signals for Ru_SA_-NiFeOOH, proving the successfully incorporation of Ru through the simple immersion method. The weak Ru XPS signal can be attributed to that Ru predominantly enriched at the catalyst layer/Ni foam interface which results in a low Ru content on the catalyst surface. The Ni 2*p* XPS spectra of Ru_SA_-NiFeOOH/Ni display peaks around 856.35 and 873.68 eV, corresponding to the Ni 2*p*_3/2_ and Ni 2*p*_1/2_ spin–orbit peaks of Ni (Ni^3+^/Ni^2+^) [35–37], respectively. As for the Fe 2*p* XPS spectra, the peaks at 712.71 and 725.81 eV can be ascribed to Fe 2*p*_3/2_ and Fe 2*p*_1/2_ spin–orbit peaks of Fe (Fe^3+^/Fe^2+^) [38–40]. The Fe binding energy in Ru_SA_-NiFeOOH shows shifts negatively compared to those in NiFeOOH, suggesting that the incorporation of Ru can alter the electronic properties of the metal sites. No detectable Cl signal is observed in either the survey spectrum of Ru_SA_-NiFeOOH, indicating the absence of Cl-containing species in the final Ru_SA_-NiFeOOH catalyst (Fig. S9). This result further rules out residual RuCl_3_ or Ru–Cl coordination in the as-prepared material.

### Electrocatalytic OER Performance in Saline Water

**Fig. 2:**
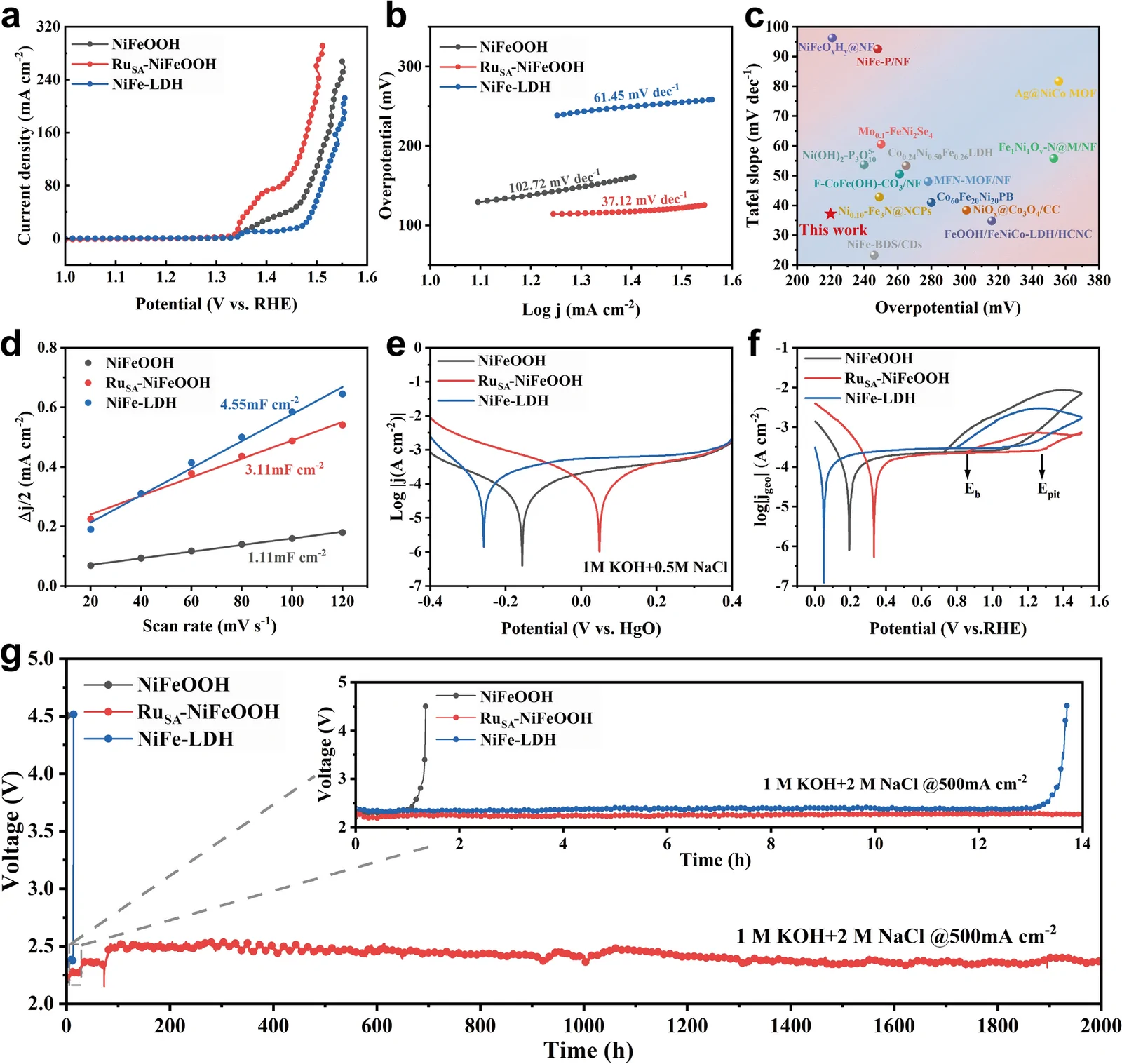
OER performances in alkaline saline electrolyte. **a** LSV curves, **b** Tafel plots, **c** Comparison of the OER overpotential at 100 mA cm^−2^ and Tafel slope of Ru_SA_-NiFeOOH with representative previously reported OER catalysts; **d** C_dl_ values; **e** Tafel plots in 1 M KOH + 0.5 M NaCl after OCP; **f** CPC plots in 1 mM KOH + 0.5 M NaCl; **g** Chronopotentiometry curve of Ru_SA_-NiFeOOH/Ni at 500 mA cm^−2^ in 1 M KOH + 2 M NaCl

The electrochemical performance of the as-prepared catalysts toward OER was evaluated using a standard three-electrode setup in 1 M KOH + 0.5 M NaCl. As shown in Fig. 2a, the OER catalytic activity of NiFeOOH/Ni is superior to that of NiFe-LDH/Ni, whose structural characterization are provided in Figs. S10 and S11, requiring only a 266-mV overpotential to drive a current density of 100 mA cm^−2^. The incorporation of Ru further reduces the OER overpotential. To optimize the Ru loading, we synthesized a series of catalysts with varied amounts of RuCl_3_ (25, 50, 100, 150, and 200 mg). Their OER activities are remarkably similar across this wide loading range, indicating no pronounced dependence on Ru content (Fig. S12 and Table S1). Based on its slightly better performance at 100 mA cm^−2^, the catalyst prepared with 50 mg RuCl_3_ was chosen for further electrochemical characterization. The optimized Ru_SA_-NiFeOOH/Ni (RuCl_3_-50 mg) demonstrates outstanding activity, requiring an overpotential of only 220 mV at 100 mA cm^−2^, far below that of NiFeOOH/Ni (266 mV) and NiFe-LDH/Ni (289 mV), highlighting the superior efficacy of the Ru single-atom modification. The Ru_SA_-NiFeOOH/Ni also shows fast OER kinetics with the lowest Tafel slope of 37.12 mV dec^−1^, compared to NiFeOOH/NF (102.72 mV dec^−1^) and NiFe-LDH/Ni (61.45 mV dec^−1^) (Fig. 2b). As shown in Fig. 2c and Table S3, our catalyst exhibits a lower overpotential at 100 mA cm^−2^ and a smaller Tafel slope compared with many previously reported OER catalysts, indicating enhanced catalytic activity and reaction kinetics. From the electrochemical impedance spectroscopy (EIS), Ru_SA_-NiFeOOH/Ni shows a smaller charge transfer resistance (*R*_ct_) and faster charge transfer behavior than other samples (Fig. S13 and Table S4). The double-layer capacitance (*C*_*dl*_) (Figs. 2d and S14) of the catalyst was measured to assess the electrochemical active surface area (ECSA) [41]. NiFe-LDH/Ni has the highest *C*_*dl*_ (4.55 mF cm^−2^) than Ru_SA_-NiFeOOH/Ni (3.11 mF cm^−2^), and NiFeOOH/Ni (1.11 mF cm^−2^), which reflects NiFe-LDH’s superior ECSA, attributable to its densely porous NiFe-LDH layer that maximizes active site density per geometric area. The ECSA-normalized LSV analysis reveals that both Ru_SA_-NiFeOOH and NiFeOOH exhibit higher intrinsic activity than NiFe-LDH (Fig. S15).

The corrosion resistance of the catalysts was evaluated in an alkaline saline electrolyte (1 M KOH + 0.5 M NaCl) using open circuit potential (OCP) measurements [42, 43]. As shown in Fig. 2e, the corrosion potentials follow the hierarchy: Ru_SA_-NiFeOOH/Ni (+ 48 mV vs. Hg/HgO) > NiFeOOH/Ni ( − 155 mV vs. Hg/HgO) > NiFe-LDH/Ni ( − 258 mV vs. Hg/HgO). Similarly, breakdown potential (*E*_*b*_), pitting potential (E_pit_) can also reflect the corrosion resistance of electrocatalysts [42, 44], therefore cyclic polarization curve (CPC) test was conducted in 1 mM KOH + 0.5 M NaCl solution (Fig. 2f). Comparative analysis reveals Ru_SA_-NiFeOOH/Ni as the most corrosion-resistant catalyst with the highest *E*_*b*_ (0.846 V vs. RHE) and *E*_pit_ (1.270 V vs. RHE), followed by NiFeOOH/Ni (*E*_*b*_ = 0.725 V, *E*_pit_ = 1.074 V), and NiFe-LDH/Ni (*E*_*b*_ = 0.788 V, E_pit_ = 1.180 V), where elevated E_b_ and E_pit_ values directly correlate with improved resistance to corrosion initiation. Notably, Ru_SA_-NiFeOOH/Ni demonstrates the smallest pitting Tafel slope, reflecting suppressed pitting propagation kinetics due to effective passivation, and exhibits the minimal hysteresis area in CPC, indicating superior re-passivation capability compared to counterparts with larger hysteresis areas that suggest irreversible localized damage. Based on the above results, Ru incorporation significantly boosts corrosion durability in the catalysts, attributed to the formation of a Ru concentrated layer on Ni surface, Ru-induced protective oxide formation, and electronic structure modification that synergistically impede both corrosion initiation and progression.

To assess the OER durability of the catalysts, initial stability tests were carried out in a standard three-electrode setup using an alkaline saline electrolyte (1 M KOH + 0.5 M NaCl) at a current density of 500 mA cm^−2^. As depicted in Fig. S16, the Ru_SA_-NiFeOOH/Ni anode, paired with a Pt foil cathode, demonstrated exceptional stability over 1000 h of electrolysis in the same electrolyte at 500 mA cm^−2^. To better approximate real industrial settings, in which continuous water evaporation leads to increasing chloride concentration, we evaluated the catalyst’s behavior in a concentrated Cl^–^ medium (1 M KOH + 2 M NaCl). The limited OER activity and quite poor durability of pristine Ni foam under alkaline conditions suggests that the significantly enhanced activity and stability observed for our catalyst arise from the introduced active phase rather than the Ni foam substrate (Fig. S17). Furthermor, as illustrated in Fig. 2g, control anodes—NiFeOOH/Ni and NiFe-LDH/Ni—suffered rapid deactivation at 500 mA cm^−2^ within only 1.2 and 13.6 h, respectively, mainly due to crack-induced substrate corrosion and mechanical failure under high current densities [24, 45]. In contrast, Ru_SA_-NiFeOOH/Ni stabilizes the catalyst–substrate interface through a Ru-rich protective layer, effectively inhibiting electrolyte penetration and delivering stable operation beyond 2000 h. These results confirm the essential role of Ru incorporation in improving resistance to Cl^−^ corrosion. ICP-OES analysis of the electrolyte after 2000 h durability test reveals only trace Ni, with Fe and Ru below the detection limit (Table S5), confirming negligible metal leaching and high catalyst stability. No visible electrode corrosion or electrolyte discoloration was observed during the 2000-h operation, and the oxygen Faradaic efficiency of the electrode at 500 mA cm^−2^ in 1 M KOH + 2 M NaCl is measured to be (98.6 ± 0.5)% (Fig. S18).

We further examined the post-test structure and morphology of Ru_SA_-NiFeOOH. SEM and TEM images confirm that the nanosheet morphology remains intact after stability testing (Figs. S19 and S20), despite an increase in surface roughness. Such roughening is indicative of electrochemical surface reconstruction, which is widely recognized to increase the electrochemically active surface area rather than cause structural degradation [46, 47]. HRTEM imaging and SAED patterns indicate a notable increase in crystallinity, with lattice fringes corresponding to the NiOOH phase. This improved crystallinity is expected to enhance electrical conductivity. XPS analysis before and after OER testing reveals significant evolution in the electronic structure. As shown in Fig. S21, the positive binding energy shifts of metal species confirm oxidation after OER. The incorporation of Ru promotes the formation of high-valence Ni active centers via electron transfer and redox buffering. This synergy not only optimizes the electronic configuration of the active sites but also helps sustain their highly active oxidation state throughout the OER process.

### Understanding the Origin of the Enhanced OER Performance

**Fig. 3:**
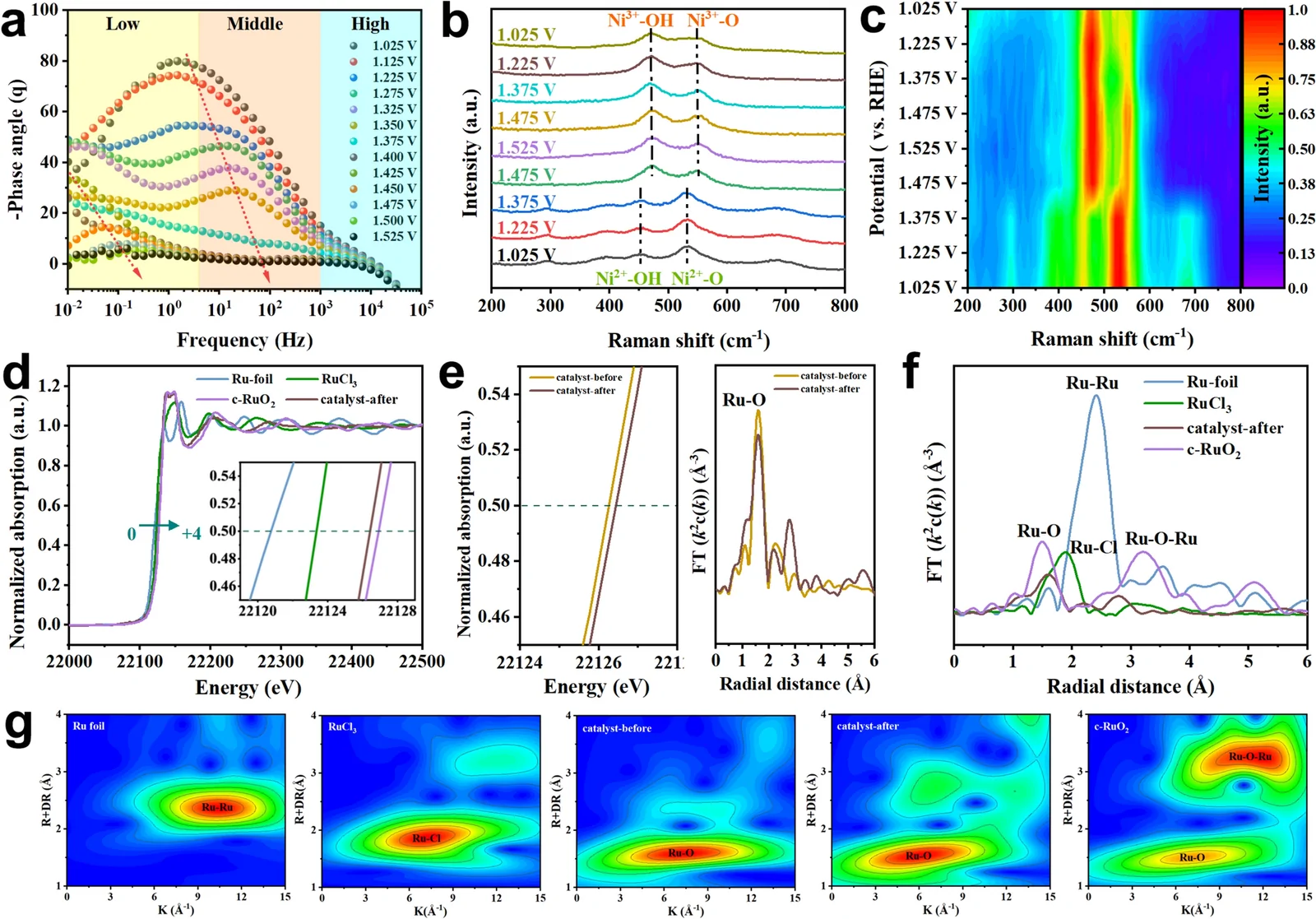
Dynamic OER process and structural evolution of Ru_SA_-NiFeOOH in alkaline saline electrolyte. **a** Bode plots of Ru_SA_-NiFeOOH for OER at different potentials in alkaline saline electrolyte; **b** In situ Raman spectra, and **c** the corresponding contour plots; **d** XANES spectra of Ru K-edge (the inset shows the enlarged absorption edge, demonstrating the Ru valance change); **e** Comparison of Ru K-edge XANES spectra and *k*^2^-weighted Fourier transform EXAFS spectra of catalysts before and after OER stability test; **f** *k*^2^-weighted Fourier transform EXAFS spectra, and **g** the corresponding wavelet transforms contour plots of catalysts before and after OER stability test, RuO_2_, RuCl_3_, and Ru foil

To elucidate the catalytic phase evolution of Ru_SA_-NiFeOOH during OER, operando EIS was conducted at a series of applied potentials. The corresponding Bode plots are presented in Figs. 3a and S22a. The decrease in phase angle within the mid-frequency range (10–10^3^ Hz) is associated with the formation of the OER-active NiOOH phase [48]. Notably, for Ru_SA_-NiFeOOH, this decline occurs at a potential 25 mV lower than that of NiFeOOH, indicating an earlier onset of phase transformation. This accelerated transition correlates with improved OER kinetics, as further supported by a more pronounced decrease in the low-frequency (10^–2^–10 Hz) phase angle. In situ Raman spectroscopy was employed to further investigate the structural evolution (Figs. 3b, c and S22b, c). Both catalysts initially display characteristic vibrational modes of Ni^2+^–OH (453 cm^−1^) and Ni^2+^–O (533 cm^−1^), corresponding to A_1g_ phonon modes [49]. For Ru_SA_-NiFeOOH, complete conversion to Ni^3^⁺ species is observed at 1.475 V versus RHE, evidenced by the emergence of Ni^3+^–OH (474 cm^−1^, e_g_ stretching vibration) and Ni^3+^–O (552 cm^−1^, A_1g_ stretching vibration) [50, 51], confirming the stabilization of the NiOOH/FeOOH active phase. In contrast, NiFeOOH exhibits no significant Raman peak shift even at 1.525 V versus RHE, suggesting sluggish oxidation from Ni^2+^ to Ni^3+^. These spectroscopic results clearly demonstrate that Ru doping facilitates the electrochemical reconstruction of the precursor into highly active NiOOH/FeOOH phases under OER conditions.

The electronic and structural evolution of Ru species in Ru_SA_-NiFeOOH after OER was also investigated using synchrotron-based XAS. Compared to the original Ru_SA_-NiFeOOH, the absorption edge energy of the catalyst after OER test is closer to that of c-RuO_2_, indicating slight change in the valence state of Ru during electrocatalysis with a calculated value of + 3.63 (Figs. 3d, e and S8). As shown in Fig. 3e, f, k^*2*^-weighted EXAFS spectra of the catalyst after test remain dominate by a Ru–O scattering path at 1.60 Å, with no discernible contributions from Ru–Ru bonds or Ru–O–Ru bonds. These results unambiguously confirm the preservation of atomically dispersed Ru species after electrochemical stability testing. This conclusion is further supported by wavelet transform analysis of the Ru K-edge EXAFS (Fig. 3g), which shows no intensity indicative of metal–metal coordination. Together, these XAS results unequivocally demonstrate the stabilization of Ru in atomic dispersion within the host structure, with no aggregation or phase separation occurring after extended electrochemical testing.

**Fig. 4:**
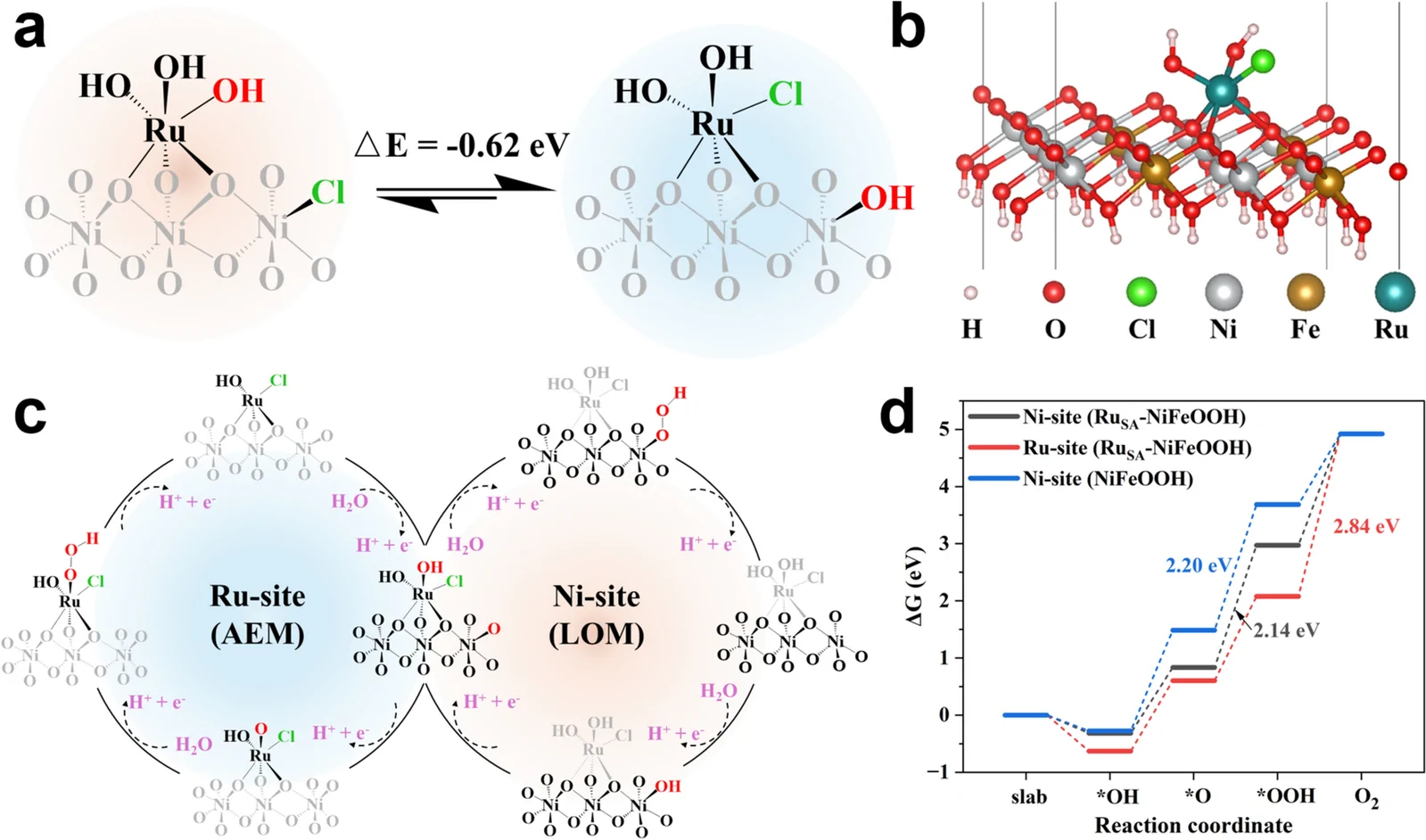
Theoretical study on the OER catalytic mechanism. **a** Scheme of the Cl-OH^–^ exchange on the loaded Ru and exposed Ni sites; **b** structural model of Ru_SA_-NiFeOOH; **c** studied OER mechanisms on Ru and Ni sites, respectively; **d** free energy diagrams on the pristine NiFeOOH and Ru_SA_-NiFeOOH

To gain mechanistic insight into the catalytic performance of Ru_SA_-NiFeOOH, density functional theory (DFT) calculations were performed. Based on experimental characterization, a monolayer (001)-facet model of NiFe-LDH with a Ni:Fe ratio of 3:1 was constructed, onto which a single Ru atom was anchored. Given that the coordination number of Ru is approximately six, the Ru atom was modeled bound to three oxygen atoms within the LDH layer, with the remaining coordination sites occupied by hydroxyl groups or Cl^−^ ions. Previous studies [52] have indicated that the OER on LDH-based catalysts proceeds via the lattice oxygen mechanism (LOM). During OER, the departure of O_2_ leaves an oxygen vacancy, which can be occupied by Cl^−^ ions from the electrolyte, leading to catalyst corrosion [43]. As shown in Figs. 4a and S23, we investigated the influence of the single Ru site on Cl^−^ adsorption at an oxygen vacancy. The Ru site exhibits a stronger affinity for Cl^−^, and a hydroxyl group on Ru spontaneously exchanges with a Cl^−^ ion at a neighboring Ni site, with an energy change of − 0.62 eV. This suggests that the presence of Ru protects exposed Ni sites from chloride-induced corrosion. Using the stable configuration illustrated in Fig. 4b, we further examined the free energy profiles of OER on Ru_SA_-NiFeOOH. Given that Ru-based systems have also been proposed as OER catalysts, two pathways were considered: the LOM pathway at the exposed Ni site and the adsorbate evolution mechanism (AEM) at the Ru site (Figs. 4c and S24). For comparison, OER on pristine NiFeOOH was also evaluated (Fig. S25). The free energy diagram in Fig. 4d shows that the potential-determining step (PDS) on pristine NiFeOOH is the formation of *OOH, with an energy barrier of 2.20 eV. With Ru incorporation, the activity at the Ni site is slightly enhanced, reducing the PDS energy to 2.14 eV. In contrast, when OER occurs at the Ru site via AEM, the desorption of *OOH is identified as the PDS, with a significantly higher energy barrier of 2.84 eV. Additionally, a recently proposed inter-metallic oxygen coupling (IMOC) mechanism [53] was evaluated for the Ru site, wherein *OOH forms via coupling of O and OH species. However, our simulations indicate that this pathway is energetically unfavorable (Fig. S26). Therefore, OER in Ru_SA_-NiFeOOH predominantly occurs at Ni sites, while the incorporated Ru primarily serves to protect adjacent Ni sites from Cl^−^ corrosion and moderately enhances their intrinsic activity.

### Stability Evaluation in Electrolyzer

**Fig. 5:**
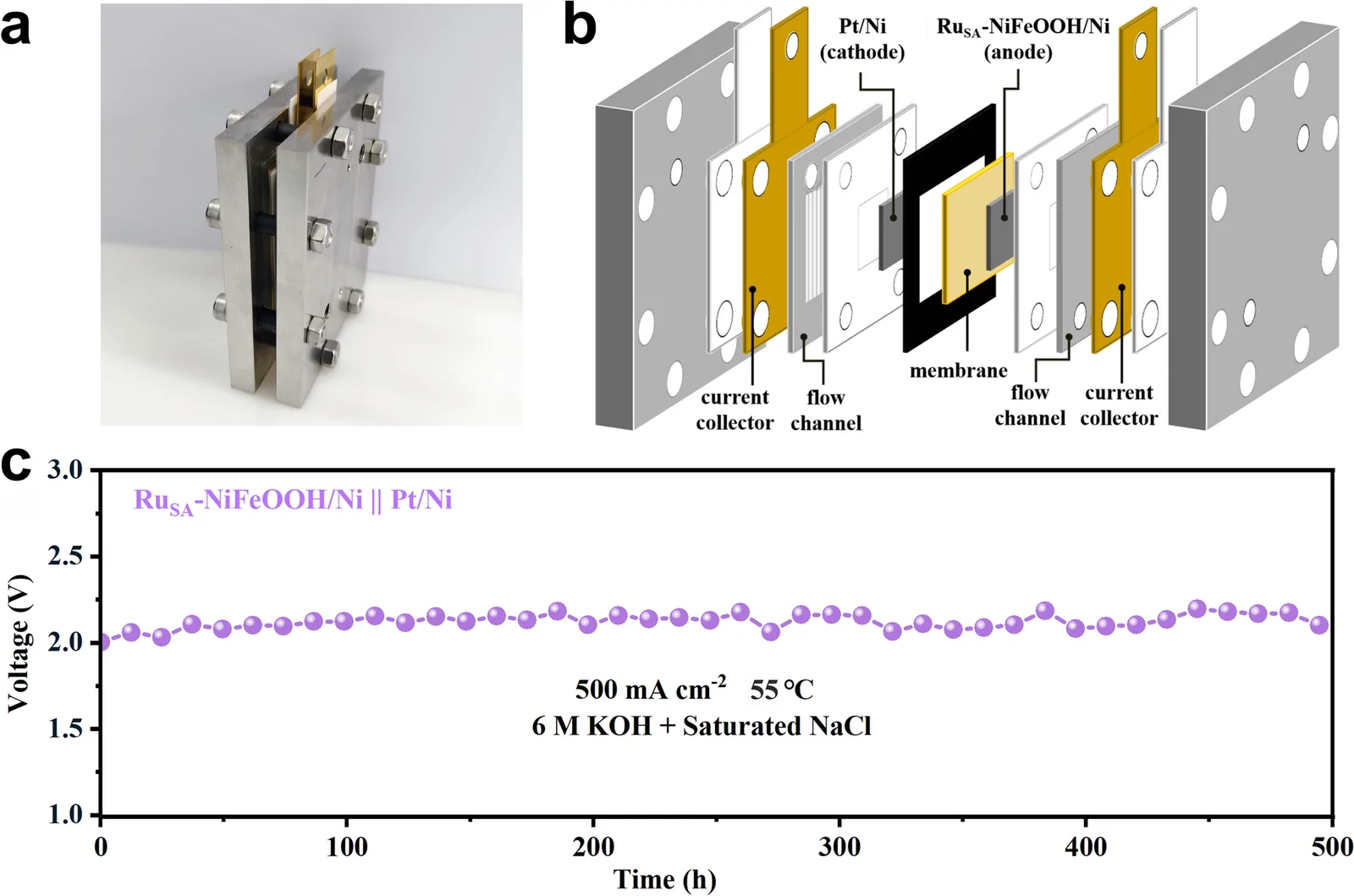
Overall water splitting performance in AEM system. **a, b** Photographs of AEM and schematic diagrams of component parts; durability test of AEM electrolyzer using Ru_SA_-NiFeOOH/Ni||Pt/Ni as electrocatalyst at 55 °C; **c** in 6.0 M KOH + saturated NaCl

A custom alkaline electrolyzer was constructed to evaluate the industrial viability of the Ru_SA_-NiFeOOH/Ni anode (Fig. 5a, b). The electrolyzer was assembled using a commercial Pt/Ni cathode and the Ru_SA_-NiFeOOH/Ni anode, with a highly concentrated alkaline saline electrolyte (6 M NaOH + saturated NaCl) to mimic industrial operating environments. The system achieved a cell voltage of approximately 2.0 V at a current density of 500 mA cm^−2^ in 6 M KOH with saturated NaCl at 55 °C and maintained stable operation for over 500 h (Fig. 5c). To further investigate the practical seawater electrolysis suitability, the electrolyte was replaced with alkaline seawater (6 M KOH + seawater). The seawater was obtained from the Beilun coast of Ningbo. It was able to maintain a low operating voltage of 1.86 V under the same operation conditions (400 mA cm^−2^, 55 °C) (Fig. S27) and remain stable for at least 500 h with a small degradation rate of 0.18 mV h^−1^. These results demonstrate that its catalytic performance and structural integrity are well preserved even in complex electrolytes, confirming that the material’s stability can be reliably extended to practical seawater environments. Moreover, the synthesis of Ru_SA_-NiFeOOH/Ni is readily scalable. A uniform electrode measuring 35 × 35 cm^2^ was successfully fabricated using the procedure described above (Fig. S28). The exceptional electrocatalytic performance and prolonged durability of the Ru_SA_-NiFeOOH/Ni electrode under industrially relevant conditions underscore its strong potential for advancing the commercialization of saline water electrolysis for hydrogen production.

## Conclusions

In summary, we demonstrate that the introduction of Ru^3+^ promotes the formation of a protective surface layer enriched with Ru atoms and a dense NiFeOOH catalyst structure, effectively blocking Cl^−^ penetration. Simultaneously, atomically dispersed ruthenium (Ru_SA_) within the NiFeOOH matrix protects active sites from Cl⁻-induced corrosion. This dual stabilization mechanism endows the Ru_SA_-NiFeOOH/Ni electrode with exceptional durability, even in highly saline environments. Specifically, it achieves outstanding stability exceeding 2000 h at an industrial current density of 0.5 A cm^−2^ in chloride-enriched alkaline electrolyte (1 M KOH + 2 M NaCl). In situ Raman and XPS analyses further reveal that Ru facilitates structural reconstruction during OER, promoting the phase evolution from Ni^2+^ to highly active Ni^3+^-enriched NiOOH/FeOOH species. Supported by DFT calculations, we confirm that Ru incorporation suppresses Cl^−^ enrichment at Ni sites. This work opens a new pathway for designing corrosion-resistant anodes in saline water electrolysis. Future efforts should focus on optimizing synthesis routes and exploring non-precious metal dopants to achieve similar dual stabilization under chloride-rich conditions.

## Supplementary Information

Below is the link to the electronic supplementary material.

Supplementary file1 (DOCX 16695 KB)
