# Supplementary material for: Unlocking Superior Stability in High-Salinity Oxygen Evolution Reaction: A Ru Stabilized NiFeOOH/Ni Anode with over 2000 h Durability
Source: Nanomicro Lett. 2026 Jan 26;18:223. doi: 10.1007/s40820-026-02072-4 (PMC12834861; doi:10.1007/s40820-026-02072-4)
Supplement: Supplementary file 1 — Supplementary file1 (DOCX 16695 KB) [file 40820_2026_2072_MOESM1_ESM.docx]

# Supporting Information for

**Unlocking Superior Stability in High-Salinity Oxygen Evolution Reaction: A Ru Stabilized NiFeOOH/Ni Anode with over 2000 h Durability**

Jin He^1,2#^, Haoyun Sheng^1,3#^, Yichao Lin^1,4*^, Bingqi Gong^1,2^, Yayun Zhao^1,4*^, Ziqi Tian^1,4^ and Liang Chen^1,4*^

^1^ Zhejiang Key Laboratory of Advanced Fuel Cells and Electrolyzers Technology, Ningbo Institute of Materials Technology and Engineering, Chinese Academy of Sciences, Ningbo, Zhejiang 315201, P. R. China

^2^ School of Materials Science and Engineering, Zhejiang University of Technology, Hangzhou, Zhejiang 3100143, P. R. China

^3^ School of Materials Science & Chemical Engineering, Ningbo University, Ningbo, Zhejiang 315211, P. R. China

^4^ University of Chinese Academy of Sciences, Beijing 100049, P. R. China

*Corresponding authors. E-mail: [yclin@nimte.ac.cn](mailto:yclin@nimte.ac.cn) (Yichao Lin); [zhaoyayun@nimte.ac.cn](mailto:zhaoyayun@nimte.ac.cn) (Yayun Zhao); [chenliang@nimte.ac.cn](mailto:chenliang@nimte.ac.cn) (Liang Chen)

**S1 Supplementary Experimental Section**

**S1.1 Materials**

Nickel nitrate hexahydrate (Ni(NO_3_)_2_·6H_2_O), hydrochloric acid (HCl), acetone (ACE), ethanol (EtOH), and sodium chloride (NaCl) were purchased from China National Pharmaceutical Group Corporation. Potassium hydroxide (KOH) was purchased from Shanghai Macklin Biochemical Technology Co., Ltd. Iron(III) nitrate nonahydrate (Fe(NO_3_)_3_·9H_2_O) was purchased from Shanghai Aladdin Biochemical Technology Co., Ltd. Ruthenium chloride (RuCl_3_) was purchased from Beijing Volk Biotechnology Co., Ltd. Nickel foam (thickness: 1.0 mm, porosity: 110 ppi) was purchased from Suzhou Sinero Technology Co., Ltd. Deionized water was prepared using OmniaPure ultra-pure water system (resistivity of 18.2 MΩ). All chemical reagents were used as received without further purification.

**S1.2 Synthesis of NiFeOOH/Ni**

NiFeOOH/Ni was synthesized following a similarprocedure to Ru_SA_-NiFeOOH/Ni, except that RuCl_3_ was omitted from the precursor solution. The precursor solution was prepared by adding 4.04 g of Fe(NO_3_)_3_·9H_2_O to 50 mL of deionized water and dissolved under stirring. Ni foams stored in ethanol were dried using mirror paper, followed by immersion in the precursor solution for 1 min, during which they were turned over at 30-second intervals. After immersion, the obtained NiFeOOH/Ni was dried in a vacuum oven at 60 °C overnight.

**S1.3 Synthesis of NiFe-LDH/Ni**

NiFe-LDH/Ni was grown on Ni foam using a hydrothermal method. Ni(NO_3_)_2_·6H_2_O (0.291 g), Fe(NO_3_)_3_·9H_2_O (0.606 g), NH_4_F (0.224 g), and urea (0.6 g) were dissolved in 35 mL deionized water under stirring. The clear solution and Ni foams were carefully transferred into a 50 mL Teflon-lined stainless-steel autoclave for solvothermal treatment at 120 ℃ for 12 h. After natural cooling to room temperature, a thin brown film formed on Ni foams, denoted as NiFe-LDH/Ni. Subsequently, NiFe-LDH/Ni was rinsed several times with deionized water and ethanol, followed by drying in a vacuum oven at 60 °C overnight.

**Note:** Ru_SA_-NiFeOOH/Ni, NiFeOOH/Ni and NiFeLDH/Ni refer to the anodes (catalysts grown on Ni foam substrate), whereas Ru_SA_-NiFeOOH, NiFeOOH and NiFeLDH denote the catalysts.

**S1.4 Electrochemical measurements**

Except for the long-term stabilization process, almost all of the electrochemical measurements were conducted using a CHI 760E electrochemical workstation ((Shanghai ChenHua, China) with our prepared samples as the working electrode (geometric area: 1 × 1 cm^2^), Pt foil (1 × 1 cm^2^) as the counter electrode and the Hg/HgO electrode as the reference electrode in a three-electrode system. Before the measurements, the samples were electrochemically activated with 50 cycles of cyclic voltammetry (CV) in 1 M KOH solution at a scan rate of 50 mV s^-1^, with potentials ranging from 0.1 to 0.6 V vs. Hg/HgO. Subsequently, linear sweep voltammetry (LSV) was performed in 1 M KOH + 0.5 M NaCl solution at a scan rate of 5 mV s^-1^, with a voltage test range of (0 - 0.9) V vs. Hg/HgO. All measured potentials were corrected with *i*R compensation (100%) and converted to reversible hydrogen electrode (RHE) scale using the following equation:E_RHE_ = E_Hg/HgO_ + 0.059 × pH + 0.098.

The stability of the electrodes was tested on a LANHE battery tester in constant charging mode. The as-prepared Ru_SA_-NiFeOOH/Ni, NiFeOOH/Ni, or NiFe-LDH/Ni was used as the anode, and Pt foil (1×1 cm^2^) served as the cathode in a two-electrode system. A peristaltic pump (300 mL h^-1^) was used to circulate the solution between an vessel and the electrolytic cell to maintain a stable electrolyte volume. The vessel was replenished with deionized water at regular intervals to maintain the solution concentration. Electrochemical impedance spectroscopy (EIS) was measured at 1.45 V vs.RHE over a frequency range of 0.01 Hz to 100 kHz, with an amplitude of 5 mV. Double-layer capacitances (C_dl_) were calculated from CV curves at different scan rates ranging from 20 mV s⁻¹ to 120 mV s⁻¹ within a non-Faraday potential range of 1.174 to 1.274 V vs. RHE. The electrochemical active surface area (ECSA) was estimated based on the following qquation: ECSA = C_dl_/C_S_, where C_S_ corresponds to the specific capacitance.

The soap-bubble method was used to determine the Faradaic efficiency by recording the evolved gas volume (mL) and the total charge passed under a constant current density of 100 mA cm^-2^. The Faradaic efficiency was calculated according to the following equation: Faraday efficiency = 4 × *F* × *V*/(1000 × *V_m_* × *I* × *t*)

where *F* is the Faraday constant (96485 C mol^-1^), *V* is the volume change of oxygen production (mL), and *V_m_* is the molar volume at ambient conditions (24.5 L mol^-1^), *I* is the applied current (A), and *t* is the electrolysis time (s).

**S1.5 Theoretical computation details**

All density functional theory (DFT) calculations were performed using the Vienna Ab initio Simulation Package (VASP) [S1]. The Perdew-Burke-Ernzerhof (PBE) functional within the generalized gradient approximation (GGA) was employed, along with the projector augmented wave (PAW) method [S2, S3]. The valence electron configurations were set as follows: O (2s, 2p), Cl (3s, 3p), Ru (5s, 4d), Ni (4s, 3d), Fe (4s, 3d), and H (1s). Spin polarization was included in the calculations, and the plane-wave cutoff energy was set to 450 eV. The convergence criteria for electronic self-consistent iterations and geometry optimization were 10^-4^ eV and 0.03 eV/Å, respectively. A first-order Gaussian smearing method with a width of 0.05 eV was applied during optimization. A single-layer (001) surface model was constructed by cleaving the bulk structure, with a vacuum slab thickness of 18 Å, and a 3 × 3 × 1 Monkhorst-Pack k-point grid was used. To account for van der Waals (vdW) interactions, the empirical Becke-Johnson D3 correction was applied [S4]. To better simulate solvation effects, the VASPsol++ nonlinear and nonlocal solvation model was employed [S5], with the bulk dielectric constant of the solvent set to 78.4 to mimic an aqueous environment. The free energy of each species was calculated in the following formula:

G = E_dft_ + E_zpe_ – TΔS

The zero-point energy and entropy correction were obtained from standard vibrational calculation, whereas the free energy of O_2_ is derived according to experimental standard formation energy of liquid water: G(O_2_) = 4.92 eV + 2G(H_2_O)– 2G(H_2_).

**S2** **Supplementary Figures and Tables**


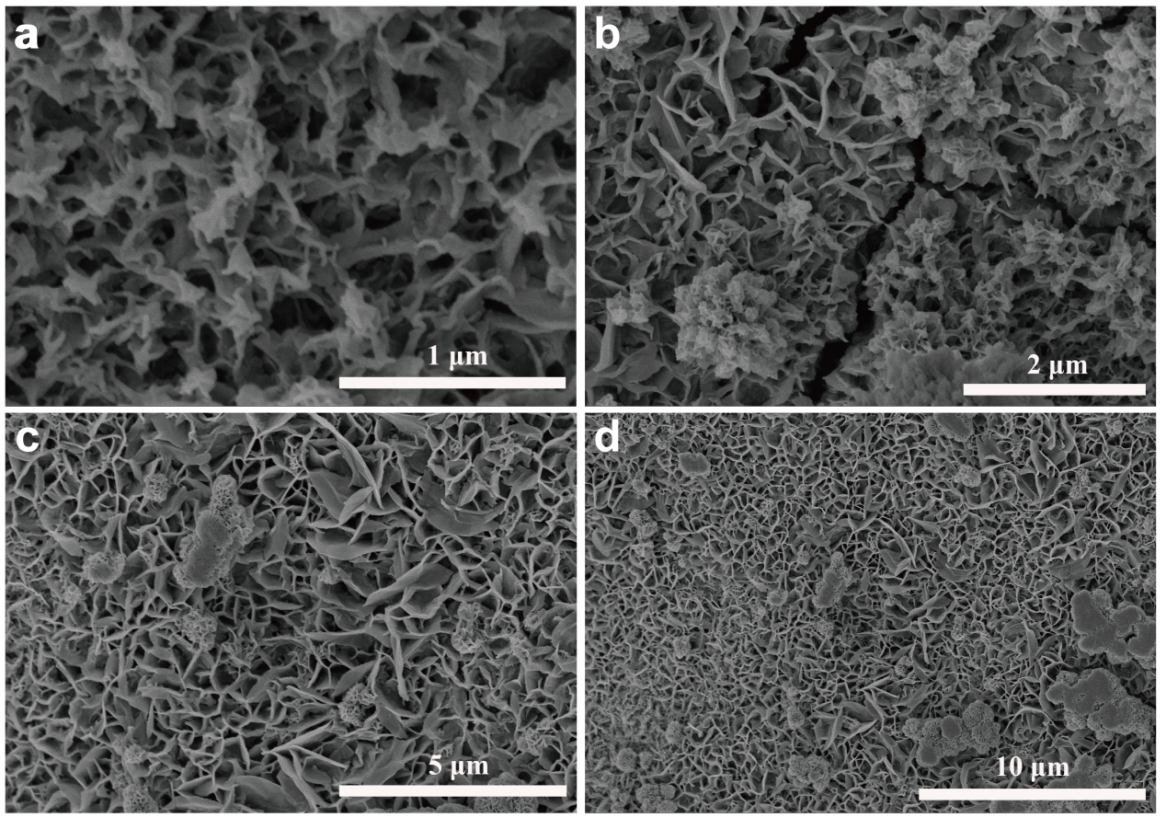


**Fig. S1** **a-d** SEM images of Ru_SA_-NiFeOOH at different magnifications


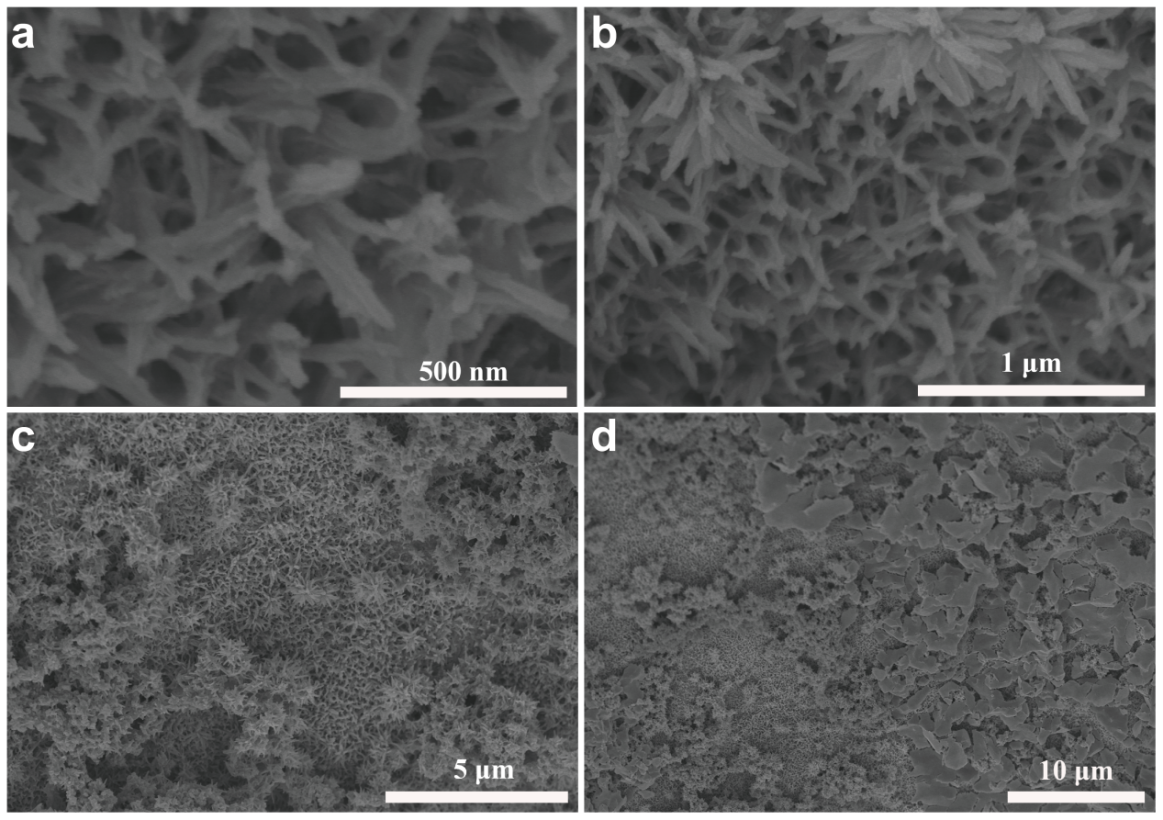


**Fig. S2** **a-d** SEM images of NiFeOOH at different magnifications


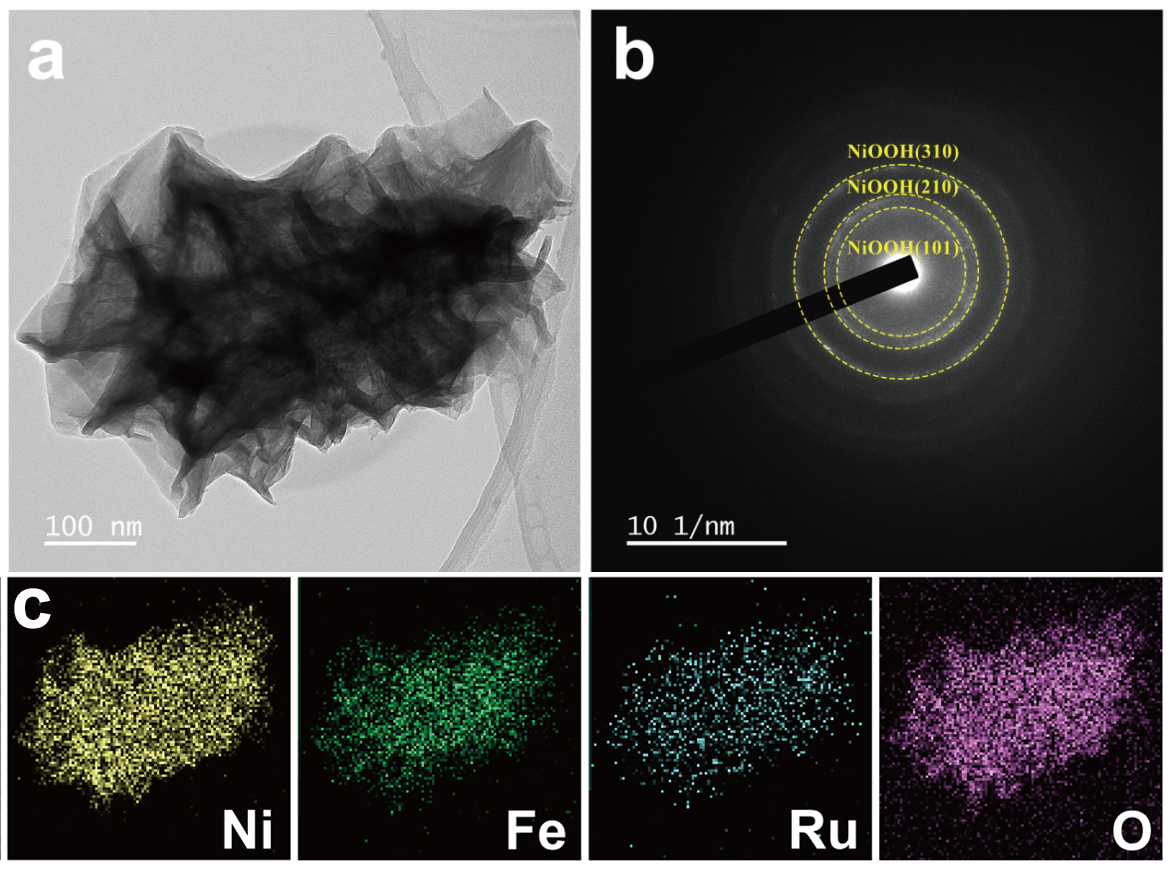


**Fig. S3 a** TEM image, **b** SAED pattern **c** EDS elemental mapping of Ru_SA_-NiFeOOH


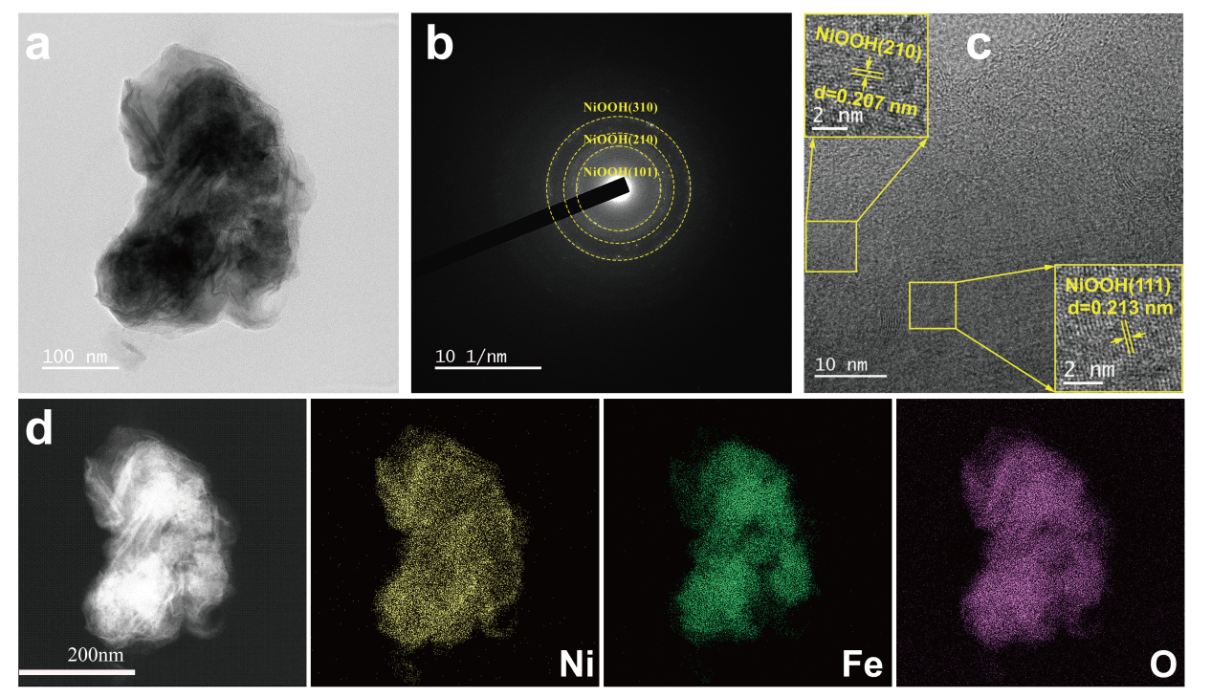


**Fig. S4** Structural characterization of NiFeOOH: **a** TEM image, **b** SAED pattern, **c** HRTEM image, and **d** EDS elemental mapping


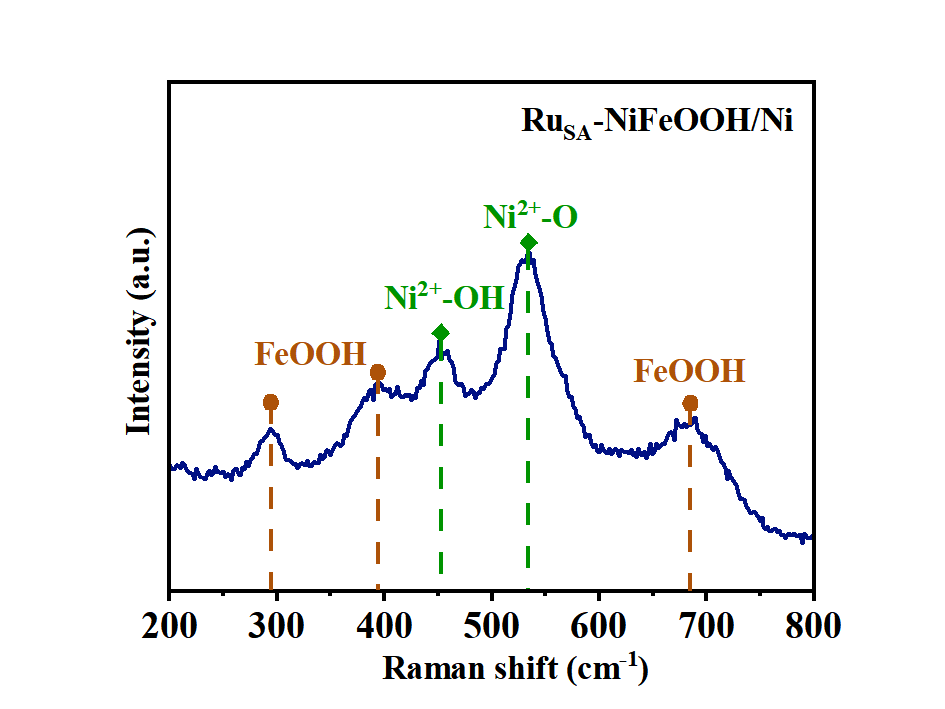


**Fig. S5** Raman spectrum of Ru_SA_-NiFeOOH


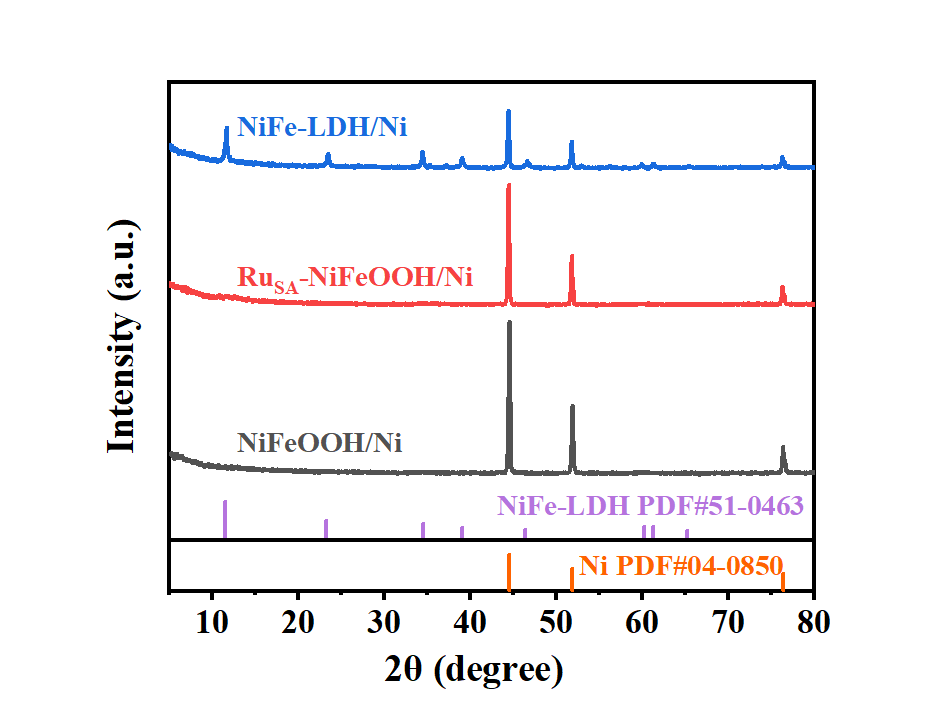


**Fig. S6** XRD patterns of NiFeOOH/Ni, Ru_SA_-NiFeOOH/Ni, and NiFe-LDH/Ni


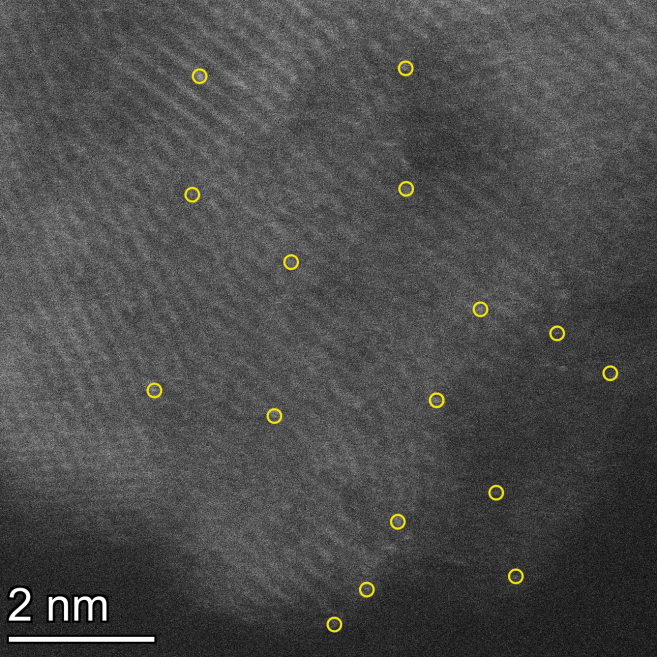


**Fig. S7** AC-HAADF STEM image of Ru_SA_-NiFeOOH, with the Ru single atoms marked with yellow circles


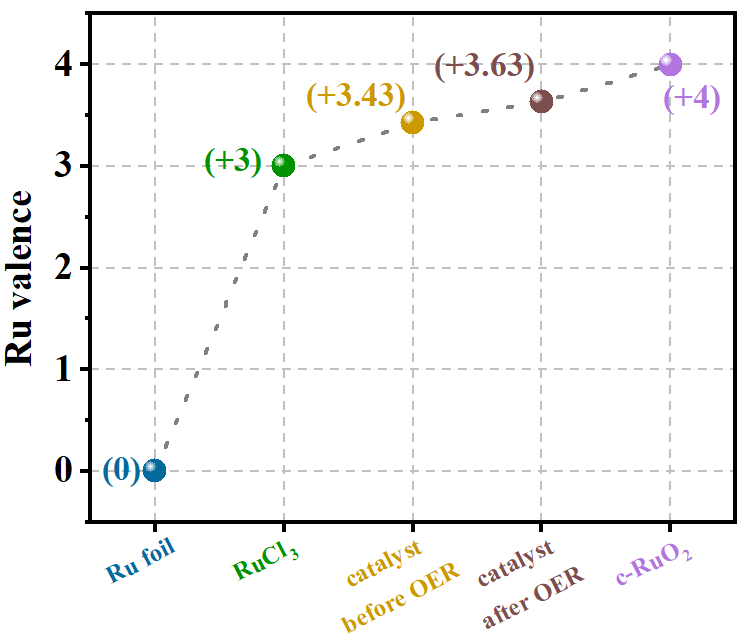


**Fig. S8** Ru atoms’ chemical valence of catalysts before and after OER stability test, RuO_2_, RuCl_3_ and Ru foil (the catalyst is Ru_SA_-NiFeOOH)


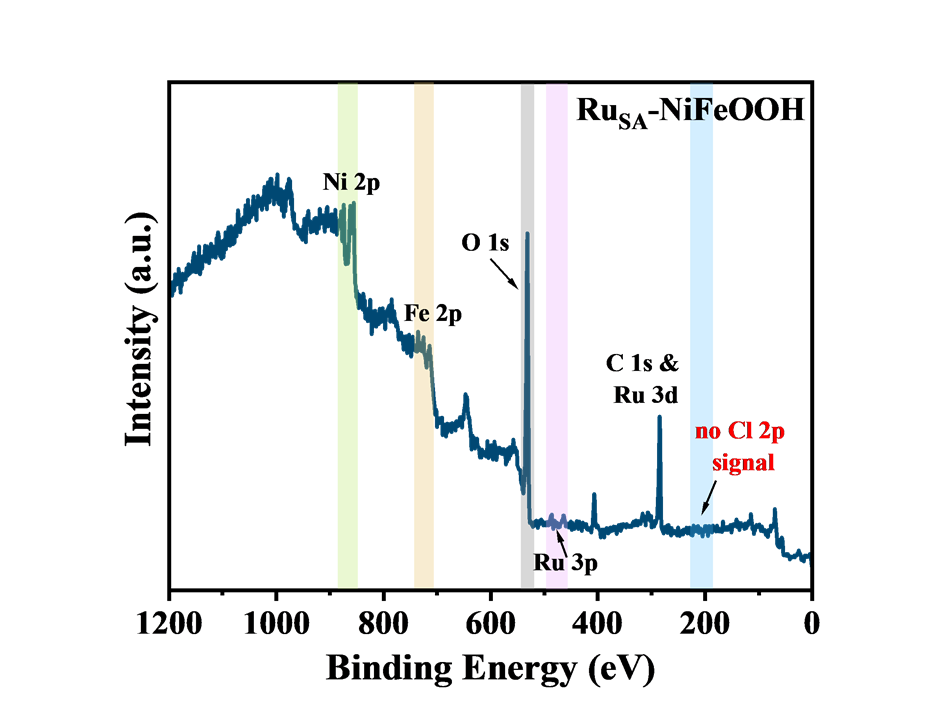


**Fig. S9** XPS survey spectrum of Ru_SA_-NiFeOOH


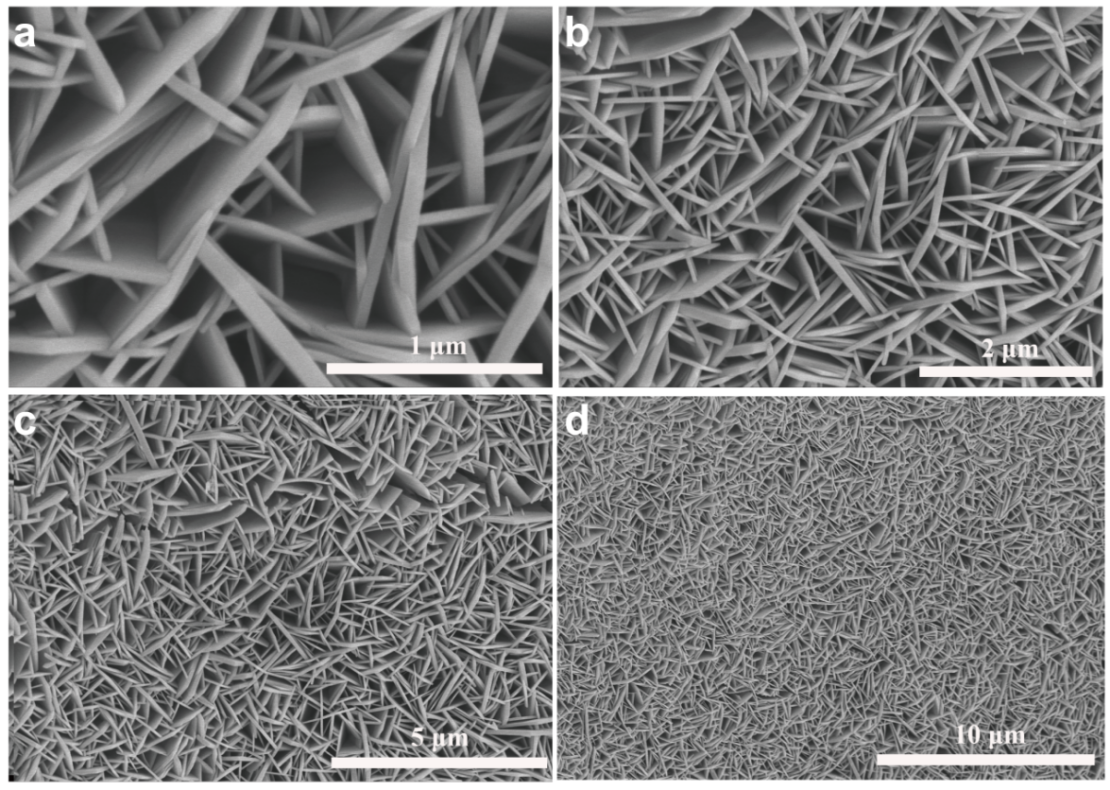


**Fig. S10** **a-d** SEM images of NiFe-LDH at different magnifications


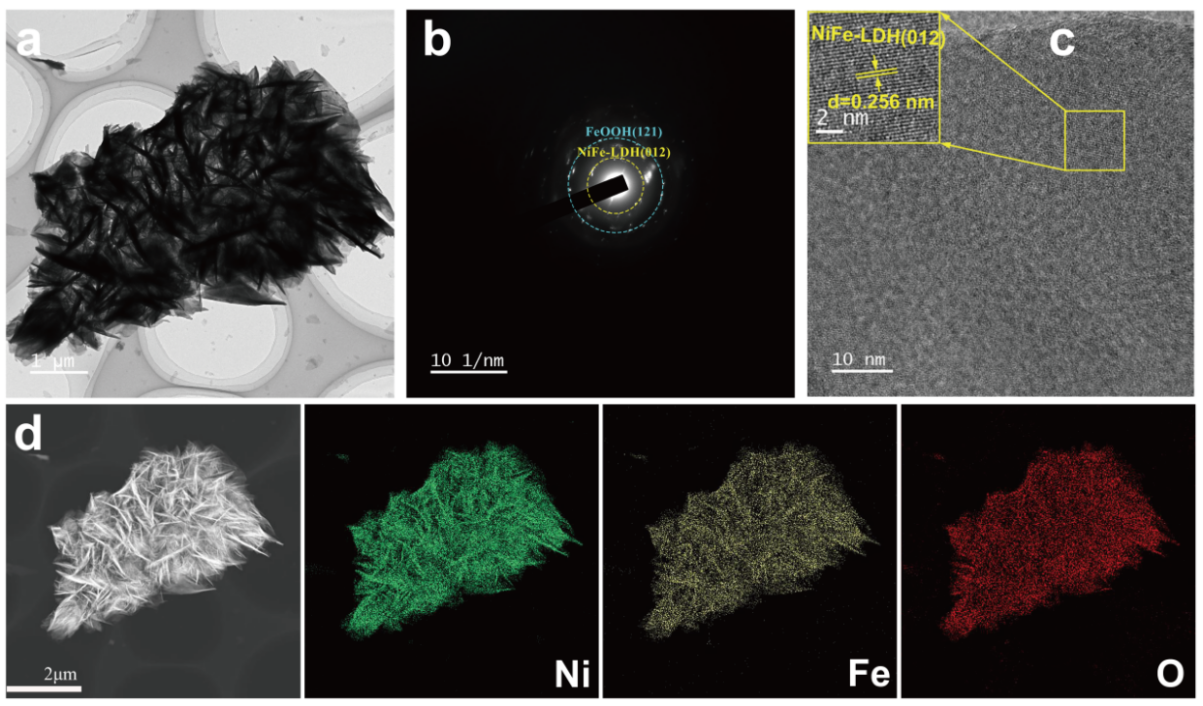


**Fig. S11** Structural characterization of NiFe-LDH: **a** TEM image, **b** SAED pattern, **c** HRTEM image, and **d** EDS elemental mapping


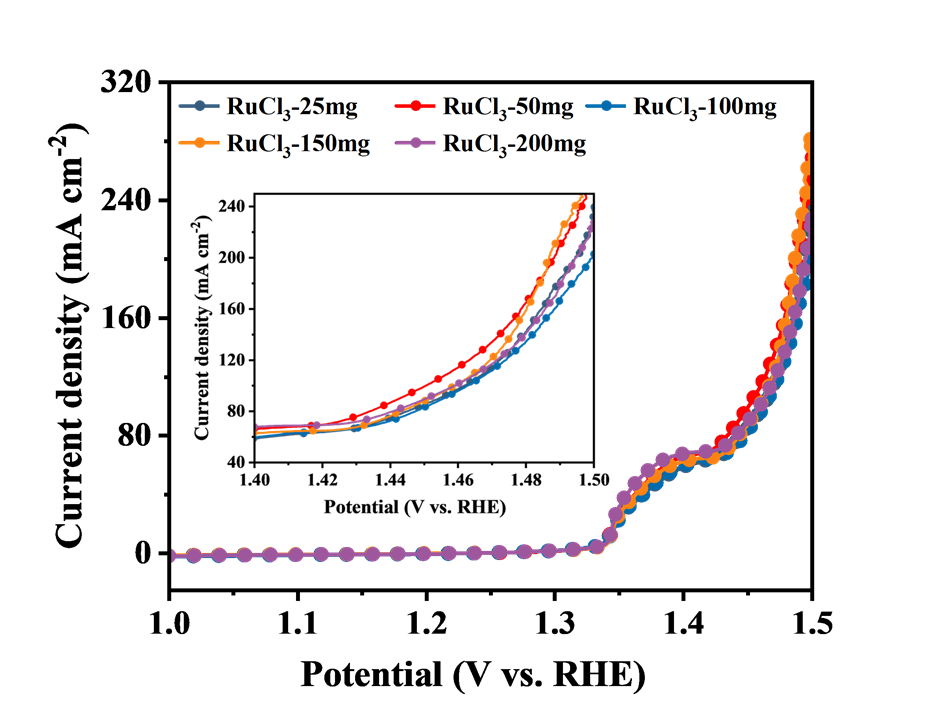


**Fig. S12** LSV curves of a series of catalysts synthesized with varying RuCl_3_ addition amounts (25 mg, 50 mg, 100 mg, 150 mg, and 200 mg) in 1 M KOH + 0.5 M NaCl


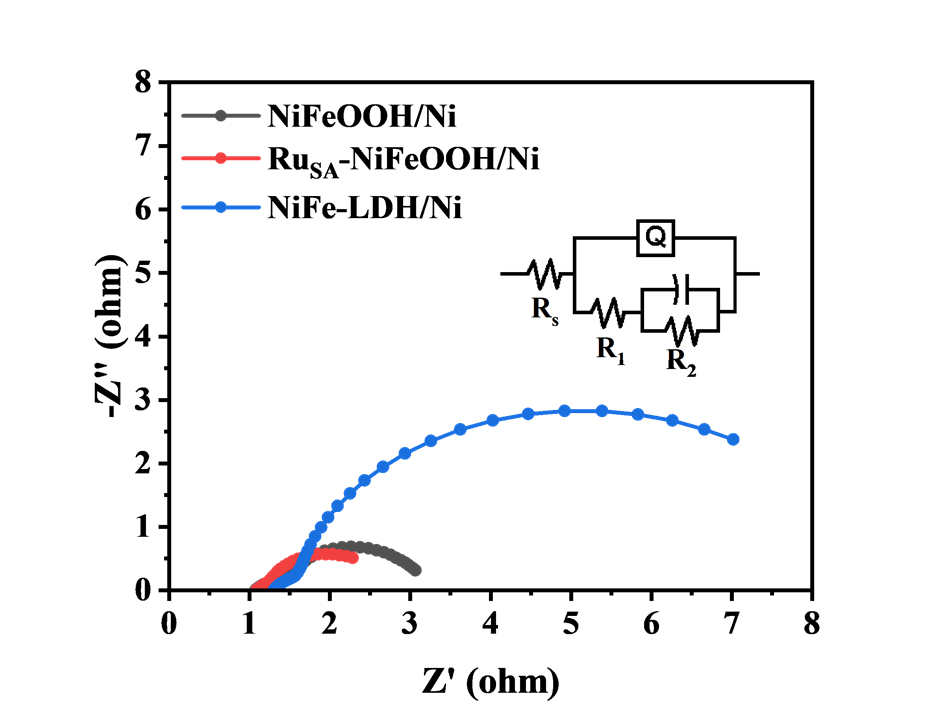


**Fig. S13** Nyquist plots of NiFeOOH/Ni, Ru_SA_-NiFeOOH/Ni, and NiFe-LDH/Ni in 1 M KOH + 0.5 M NaCl. (Equivalent circuit fitting parameters are shown in Table S4.)


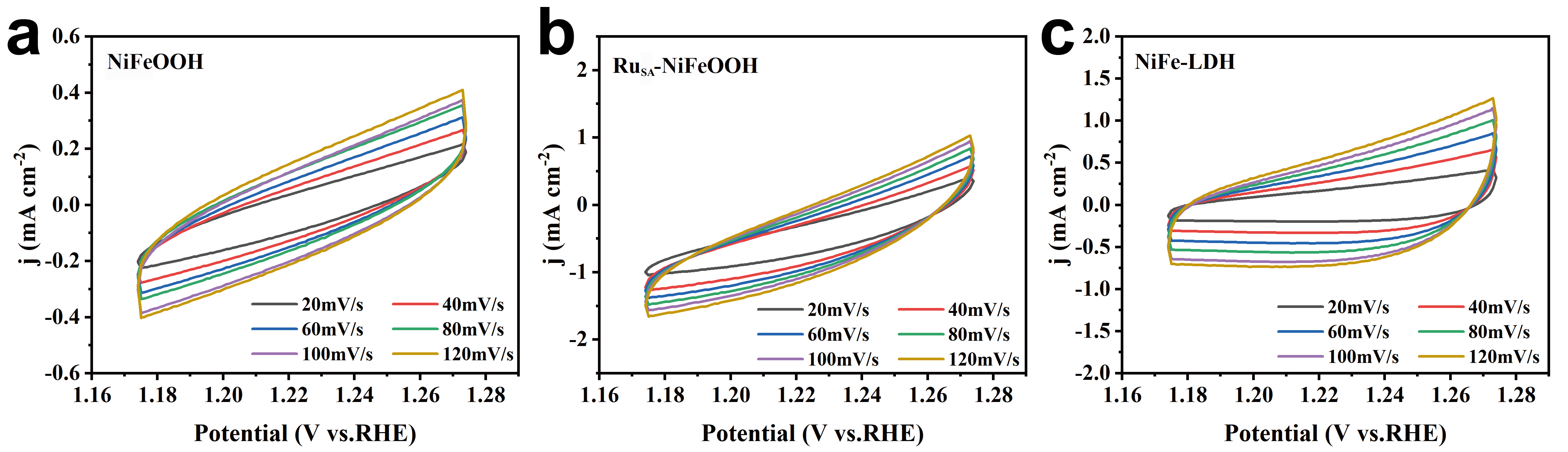


**Fig. S14** Cyclic voltammetry profiles of **a** NiFeOOH/Ni, **b** Ru_SA_-NiFeOOH/Ni, and **c** NiFe-LDH/Ni acquired at varying scan rates (20 - 120 mV·s^-1^) under a potential window of 1.174 - 1.274 V (vs. RHE) in 1 M KOH + 0.5 M NaCl electrolyte


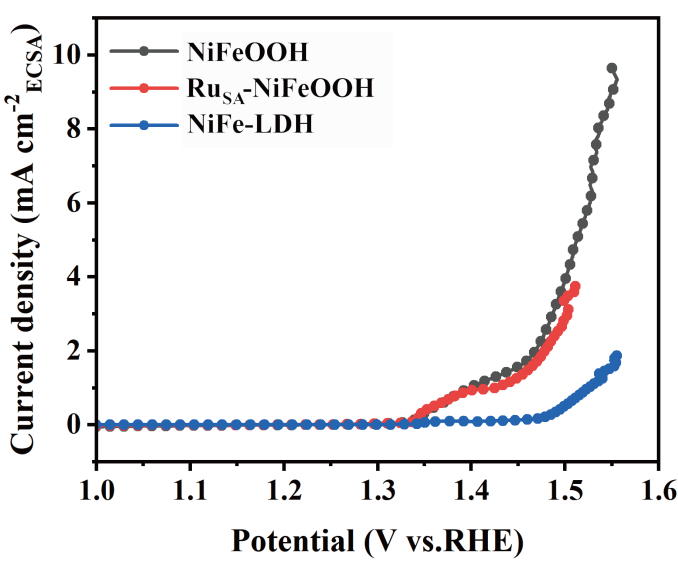


**Fig. S15** Polarization curves normalized by ECSA of electrodes


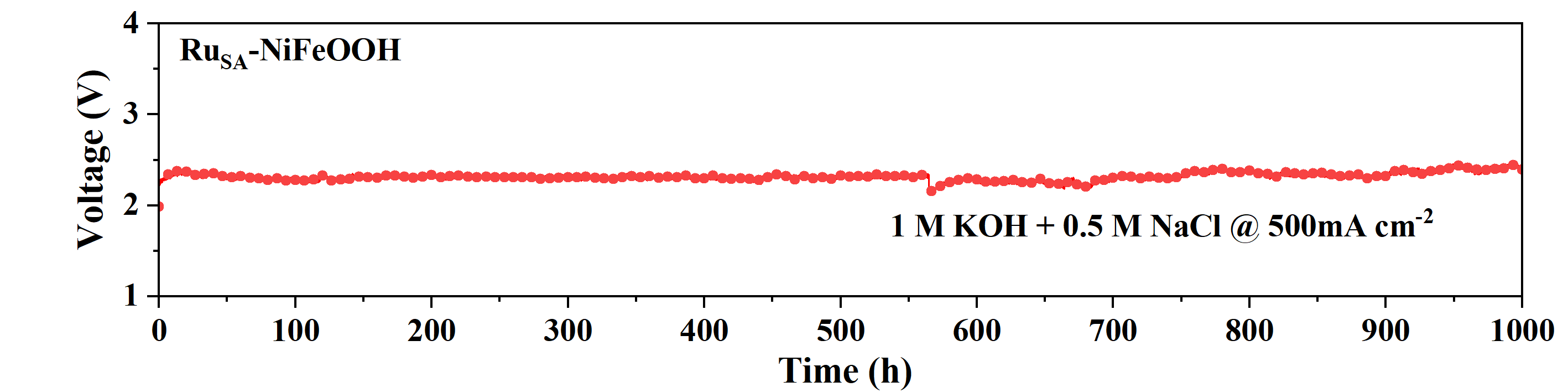


**Fig. S16** Chronpotentiometry curve of Ru_SA_-NiFeOOH/Ni at 500 mA cm^-2^ in 1 M KOH + 0.5 M NaCl for 1000 h (two-electrode test)


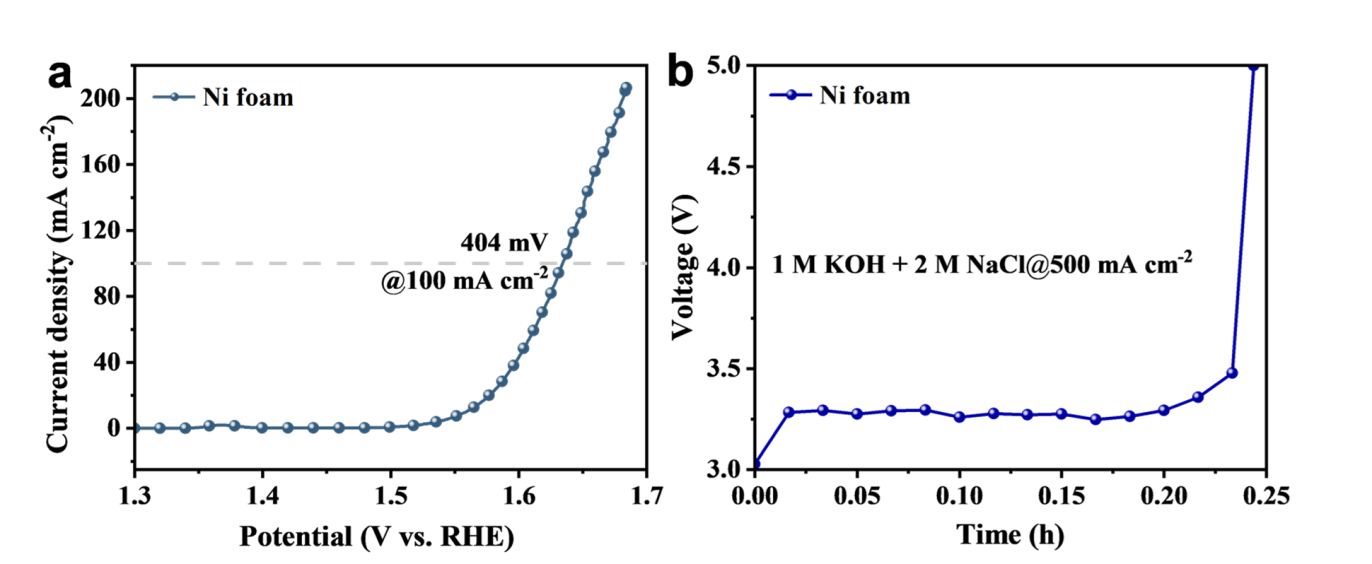


**Fig. S17** **a** LSV curves of Ni foam and **b** Chronpotentiometry curve of Ni foam at 500 mA cm^-2^ in 1 M KOH + 2 M NaCl


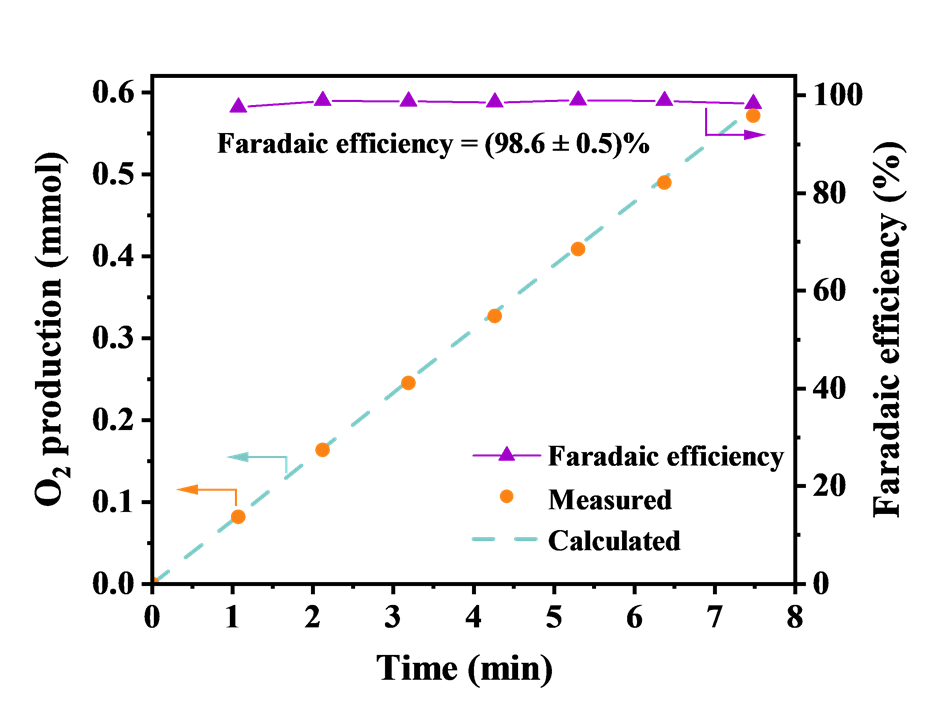


**Fig. S18** Faradaic efficiency values of Ru_SA_-NiFeOOH/Ni and experimentally measured O_2_ gas versus time at 500 mA cm^-2^ in 1M KOH + 2 M NaCl


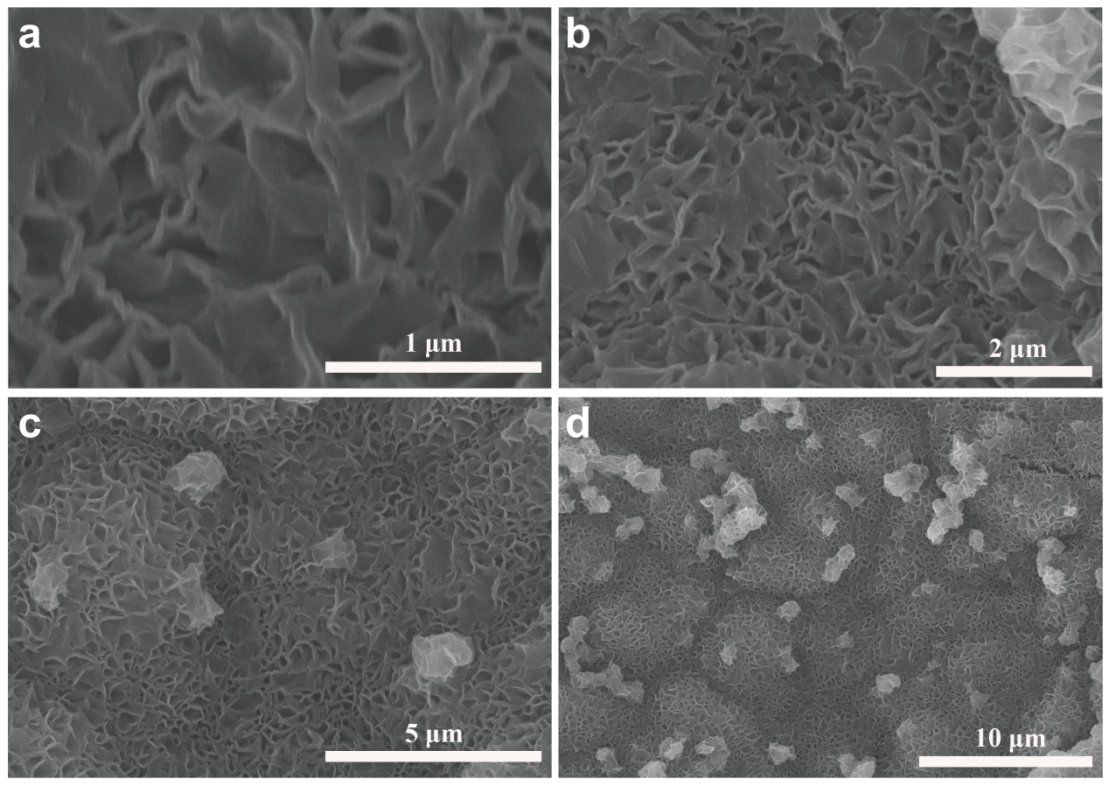


**Fig. S19** **a-d** SEM images of Ru_SA_-NiFeOOH/Ni after stability test at different magnifications


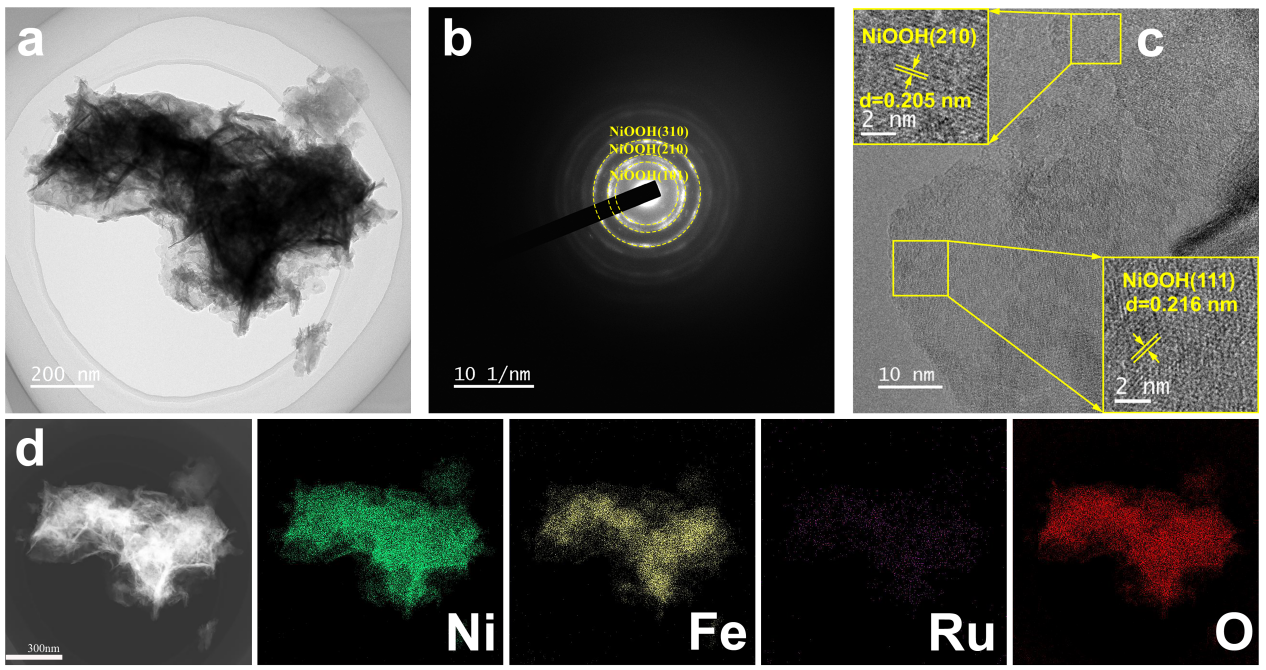


**Fig. S20** Structural characterization of Ru_SA_-NiFeOOH/Ni after stability test: **a** TEM image, **b** SAED pattern, **c** HRTEM image, and **d** EDS elemental mapping


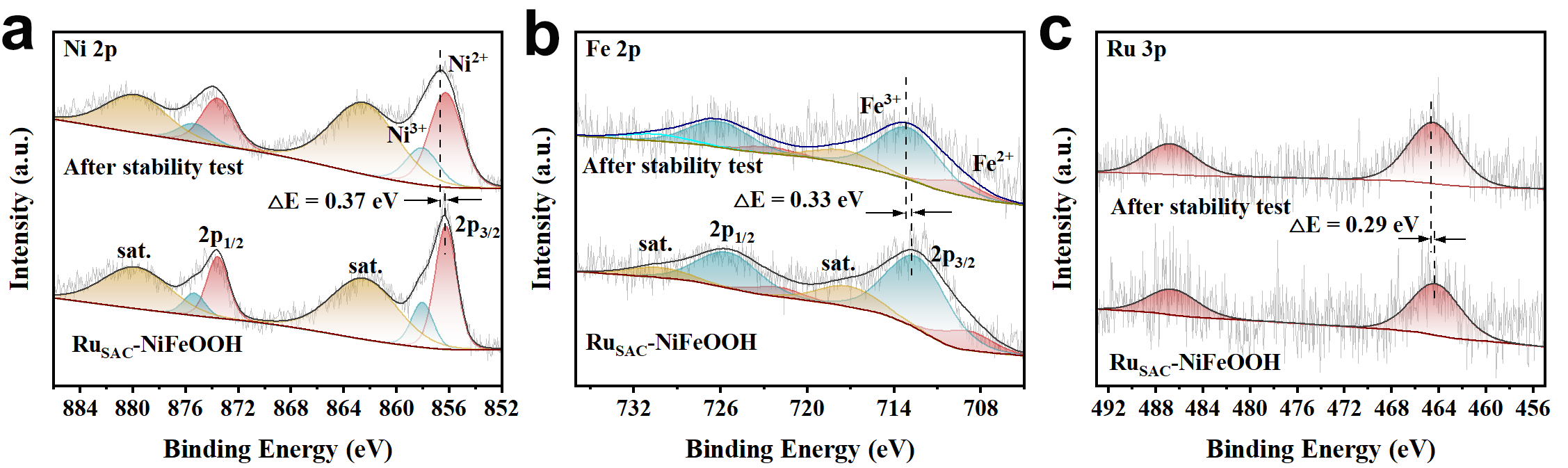


**Fig. S21** High-resolution XPS peaks of **a** Ni 2p, **b** Fe 2p, and **c** Ru 3p for Ru_SA_-NiFeOOH/Ni before and after OER stability test


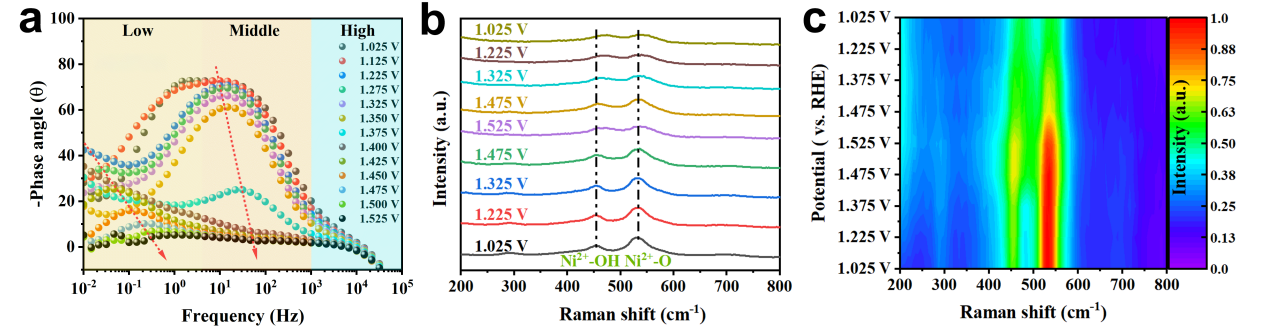


**Fig. S22** **a** Bode plots of NiFeOOH for OER at different potentials in alkaline saline electrolyte; **b** In situ Raman spectra, and **c** the corresponding contour plots of NiFeOOH


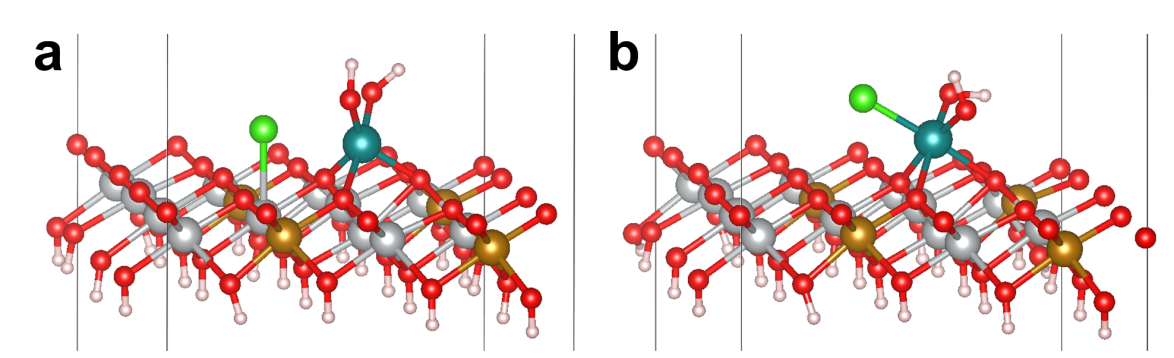


**Fig. S23** Comparison of Ru_SA_-NiFeOOH (001) models **a** before and **b** after optimizing Cl^–^ adsorption on Ni sites


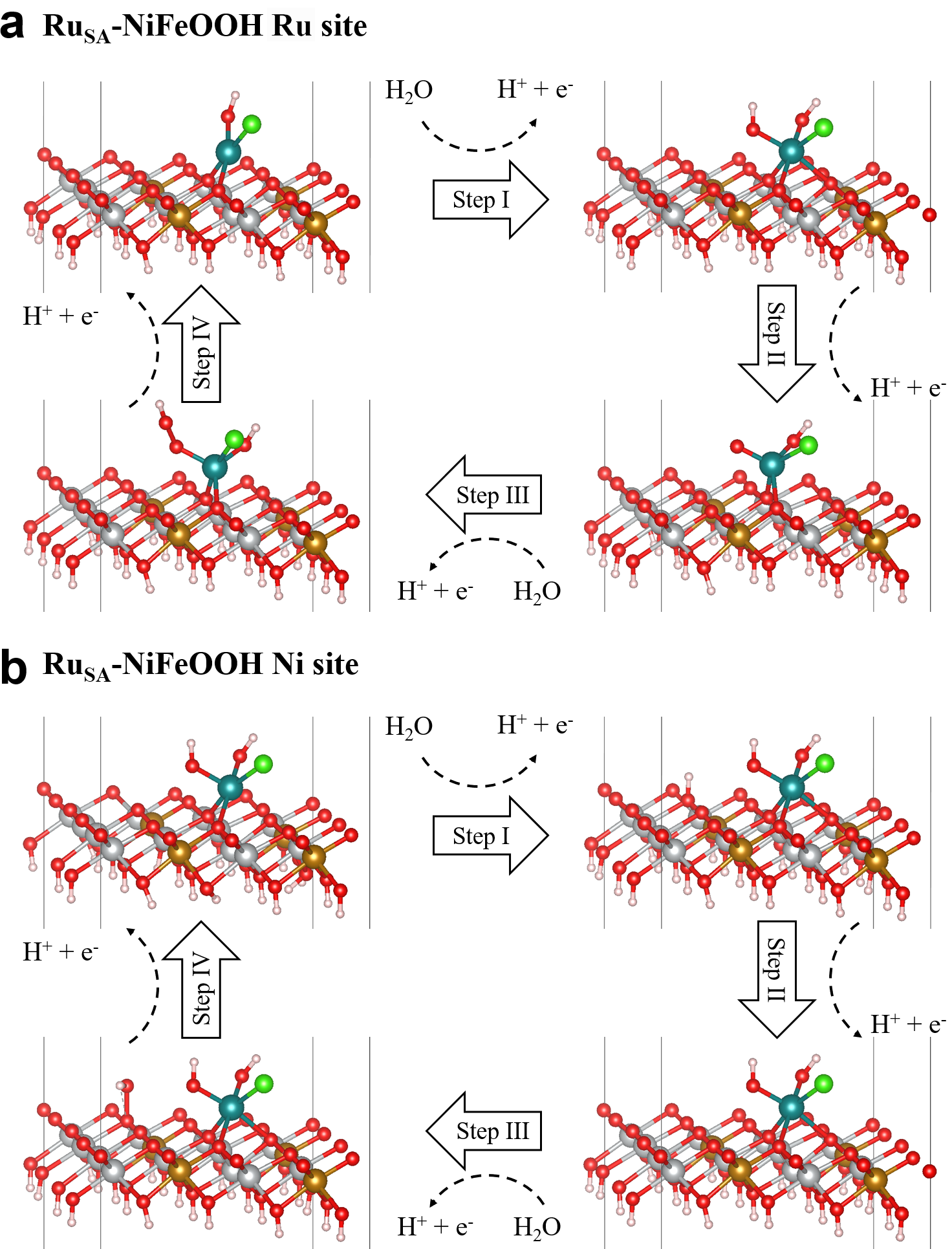


**Fig. S24** Structural models of the OER reaction intermediates for **a** Ru sites and **b** Ni sites in Ru_SA_-NiFeOOH


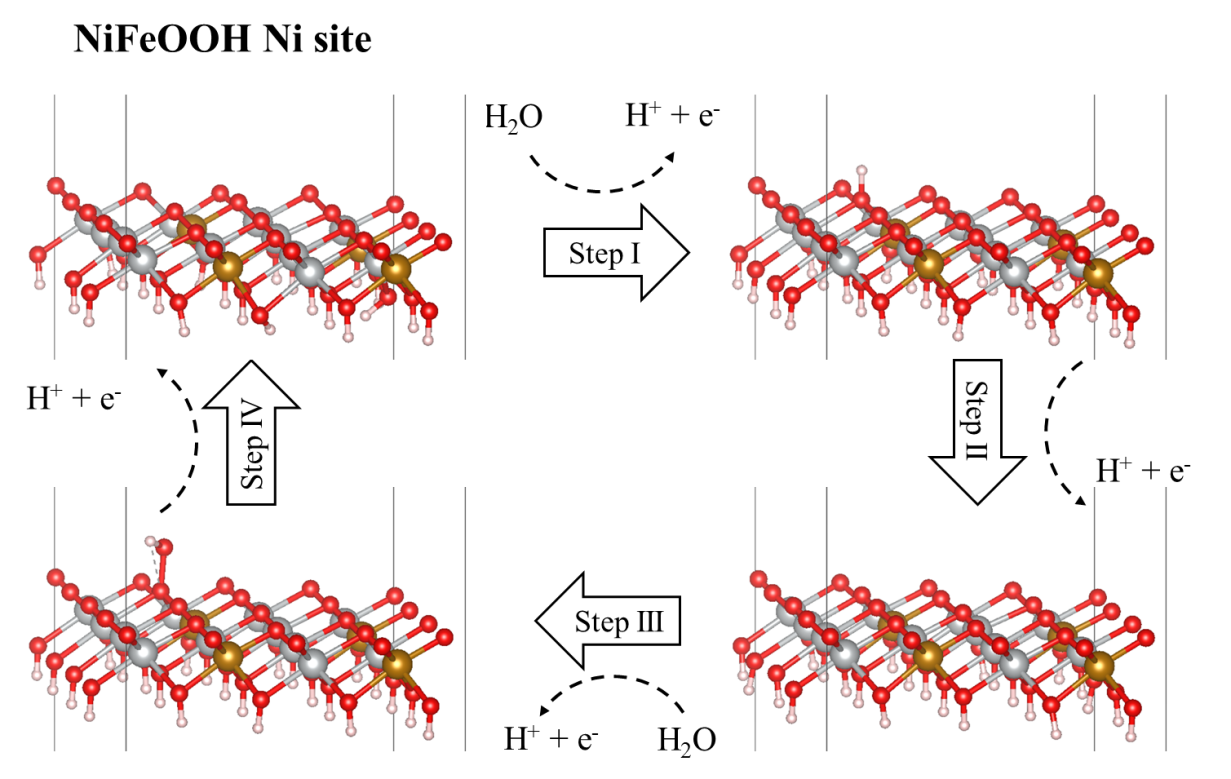


**Fig. S25** Structural models of the OER reaction intermediates Ni sites in NiFeOOH


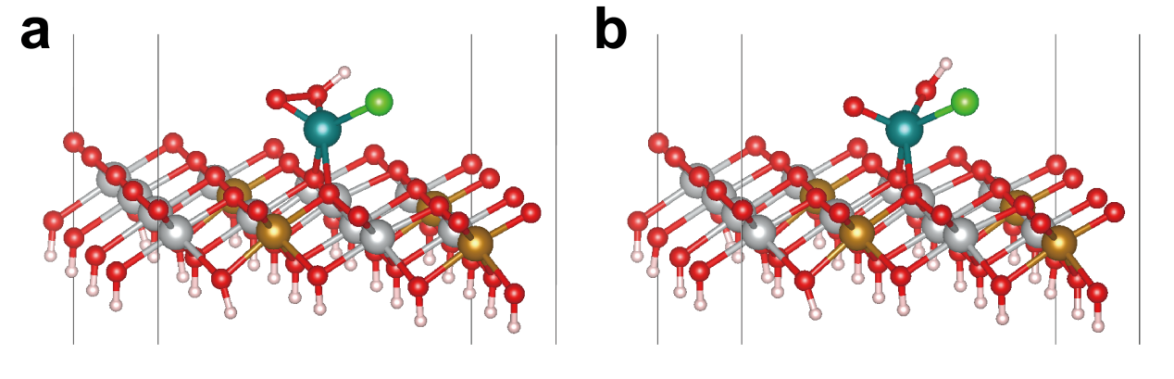


**Fig. S26** **a** Before optimization of O and OH coupling on Ru atoms, **b** after optimization of O and OH coupling on Ru atoms


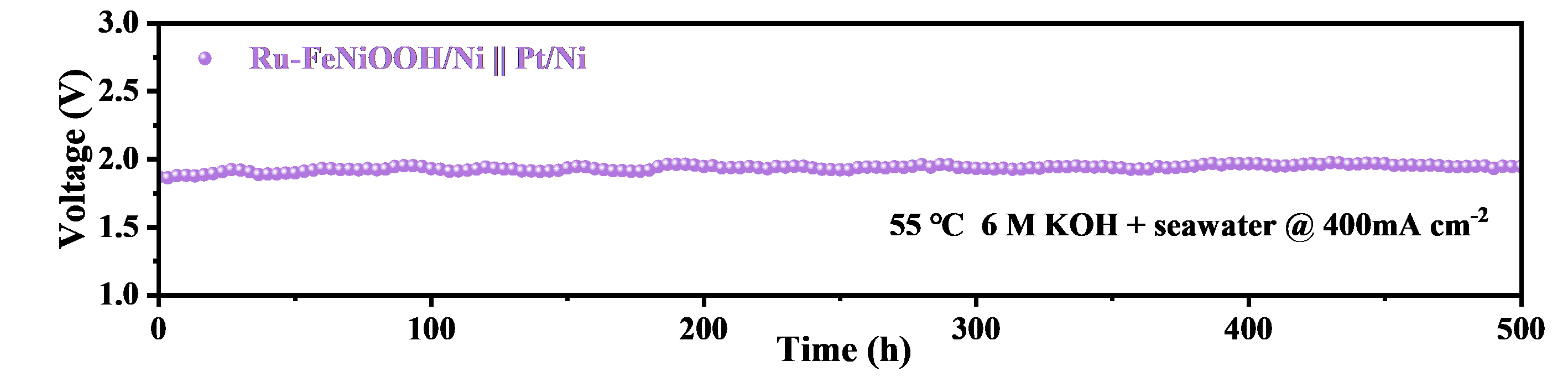


**Fig. 27** Durability test of AEM electrolyzer using Ru_SA_-NiFeOOH/Ni||Pt/Ni as electrocatalyst at 55 °C in 6.0 M KOH + seawater


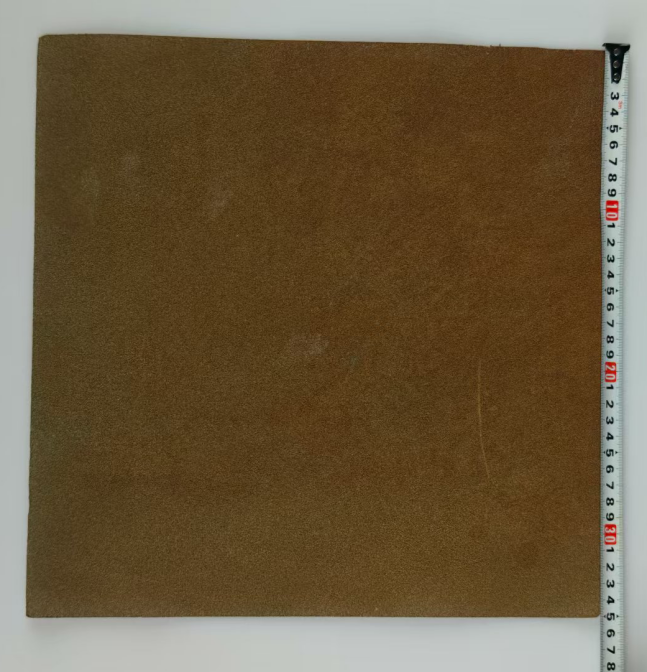


**Fig. S28** Ru_SA_-NiFeOOH/Ni with a large area of 35 × 35 cm^2^

**Table S1** ICP-OES analysis of NiFeOOH and Ru_SA_-NiFeOOH

| Samples | Amount of RuCl_3_ added | Ni (Atom%)*^a^* | Fe (Atom%)*^a^* | Ru (Atom%)*^a^* |
| --- | --- | --- | --- | --- |
| NiFeOOH | 0 mg | 83.30 | 16.70 | / |
|  | 25 mg | 75.02 | 24.62 | 0.35 |
| Ru_SA_-NiFeOOH | 50 mg | 73.01 | 26.37 | 0.62 |
|  | 100 mg | 74.66 | 24.24 | 1.10 |
| *^a^* Atom% relative to the total metal atoms. | | | | |

**Table S2** Fitting parameters of Ru_SA_-NiFeOOH catalyst (S_0_^2^=0.60 from Ru foil)

| Sample | Path | C.N.*^a^* | R (Å)*^b^* | σ^2^ × (10^-3^ Å^2^)*^c^* | R factor*^d^* |
| --- | --- | --- | --- | --- | --- |
| Ru_SA_-NiFeOOH | Ru-O | 5.9 ± 0.3 | 2.06 ± 0.01 | 3.0 | 0.011 |
| *^a^* CN is the coordination number *^b^* R is the distance between the absorber-scatterer path  *^c^* σ^2^ is the Debye-Waller (disorder factor) *^d^* R factor is a measure of the goodness of fit | | | | | |

**Table S3** Performance comparison of the Ru_SA_-NiFeOOH/Ni catalyst with recently reported high-performance OER catalysts in saline water

| Samples | Overpotentials  (mV) @100 mAcm^-2^ | Tafel slope  (mV dec^-1^) | Refs. |
| --- | --- | --- | --- |
| Ru_SA_-NiFeOOH/Ni | 220 | 37.12 | This work |
| FeNiO_v_-N@M/NF | 353 | 55.8 | [S6] |
| FeOOH/FeNiCo-LDH/HCNC | 316 | 34.8 | [S7] |
| NiFe-BDC/CDs | 246 | 23.3 | [S8] |
| Mo_0.1_-FeNi_2_Se_4_ | 250 | 60.6 | [S9] |
| NFN-MOF/NF | 278 | 48 | [S10] |
| NiO_x_@Co_3_O_4_/CC | 301 | 38.4 | [S11] |
| F-CoFe(OH)-CO_3_/NF | 261 | 50.51 | [S12] |
| NiFeO_x_H_y_@NF | 221 | 96.2 | [S13] |
| NiFe-P/NF | 248 | 92.5 | [S14] |
| Ni(OH)_2_-P_3_O_10_^5-^ | 240 | 53.7 | [S15] |
| Co_60_Fe_20_Ni_20_PB | 280 | 41 | [S16] |
| Ag@NiCoMOF | 356 | 81.7 | [S17] |
| Ni_0.10_-Fe_3_N@NCPs | 249 | 42.8 | [S18] |
| NiFe-Co_2_(OH)_3_Cl | 369 | 49.9 | [S19] |

**Table S4** Equivalent circuit fitting parameters obtained from EIS measurements using the model shown in Fig. S13.

| Samples | R_st_ (Ω) | CPE (S·sec^n^) | Freq. power, n | R_ct_ (Ω) | Capacitance (F) | R_f_ (Ω) |
| --- | --- | --- | --- | --- | --- | --- |
| Ru_SA_-NiFeOOH/Ni | 1.09 | 1.21 | 0.65 | 0.40 | 1.52 | 1.35 |
| NiFeOOH/Ni | 1.04 | 0.38 | 0.48 | 0.79 | 0.67 | 1.72 |
| NiFeLDH/Ni | 1.32 | 0.33 | 0.71 | 0.71 | 0.39 | 7.01 |

**Table S5** Metal concentrations in the electrolyte after a 2000-hour durability test

| Sample | Test condition (electrolysis) | Ni (mg/L) | Ru (mg/L) | Fe (mg/L) |
| --- | --- | --- | --- | --- |
| Ru_SA_-NiFeOOH/Ni | 500 mA cm^-2^, 2000 h, 1.0 M KOH + 2 M NaCl | 0.01 | ND | ND |
| Note: Metal concentrations were quantified by ICP-OES after 2000-hour durability test. analysis. Values below the detection limit are reported as “ND”. | | | | |

# Supplementary References

1. G. Kresse, J. Furthmüller, Efficient iterative schemes for *ab initio* total-energy calculations using a plane-wave basis set. Phys. Rev. B **54**(16), 11169–11186 (1996). <https://doi.org/10.1103/physrevb.54.11169>
2. J.P. Perdew, K. Burke, M. Ernzerhof, Generalized gradient approximation made simple. Phys. Rev. Lett. **77**(18), 3865–3868 (1996). <https://doi.org/10.1103/physrevlett.77.3865>
3. P.E. Blöchl, Projector augmented-wave method. Phys. Rev. B **50**(24), 17953–17979 (1994). <https://doi.org/10.1103/physrevb.50.17953>
4. S. Grimme, S. Ehrlich, L. Goerigk, Effect of the damping function in dispersion corrected density functional theory. J. Comput. Chem. **32**(7), 1456–1465 (2011). <https://doi.org/10.1002/jcc.21759>
5. S.M. Rezwanul Islam, F. Khezeli, S. Ringe, C. Plaisance, An implicit electrolyte model for plane wave density functional theory exhibiting nonlinear response and a nonlocal cavity definition. J. Chem. Phys. **159**(23). 234117 (2023). <https://doi.org/10.1063/5.0176308>
6. R.M. Bhattarai, L. Nguyen, N. Le, K. Chhetri, D. Acharya et al., Cyanide functionalization and oxygen vacancy creation in Ni-Fe nano petals sprinkled with MIL-88A derived metal oxide nano droplets for bifunctional alkaline seawater electrolysis. Small **21**(30), 2410027 (2025). <https://doi.org/10.1002/smll.202410027>
7. Y. Luo, Y. Yang, Y. Tian, Q. Wu, W.-F. Lin et al., Collaborative reconstruction of FeOOH/FeNiCo-LDH heterogeneous nanosheets for enhancing anion exchange membrane seawater electrolysis. J. Mater. Chem. A **13**(10), 7136–7148 (2025). <https://doi.org/10.1039/d4ta08586h>
8. Z. Tang, D. Chen, W. Li, H. Li, J. Tu et al., Enhanced oxygen evolution reaction through improved lattice oxygen activity *via* carbon dots incorporation into MOFs. J. Colloid Interface Sci. **685**, 361–370 (2025). <https://doi.org/10.1016/j.jcis.2025.01.133>
9. X. Zhang, Z. Wang, S. Cao, X. Lin, X. Chen et al., Breaking the scaling relationship for high-performance seawater oxidation through lattice distortion triggered by molybdenum. J. Mater. Sci. Technol. **225**, 165–173 (2025). <https://doi.org/10.1016/j.jmst.2024.10.050>
10. Y. Hu, X. Zhao, Y. Min, Q. Xu, Q. Li, An *in situ* grown NiFe-based MOF for efficient oxygen evolution in alkaline seawater at high current densities. New J. Chem. **49**(7), 2665–2673 (2025). <https://doi.org/10.1039/d4nj05248j>
11. H. Peng, X. Zhang, B. Wang, Y. Cao, M. wang et al., Optimizing seawater electrolysis with electronically tuned Co_3_O_4_-NiO*_x_* heterostructures. Appl. Surf. Sci. **686**, 162162 (2025). <https://doi.org/10.1016/j.apsusc.2024.162162>
12. T. Zheng, J. Shi, D. Wang, Y. Min, Q. Xu et al., CoFe hydroxide nanospheres for enhanced alkaline water splitting and seawater oxidation: anion doping effects of fluorine and carbonate. Chem. **31**(5), e202403628 (2025). <https://doi.org/10.1002/chem.202403628>
13. J. Zhang, X. Ji, C. Han, Z. Li, S. Jiang et al., Amorphous/crystalline Ni-Fe based electrodes with rich oxygen vacancies enable highly active oxygen evolution in seawater electrolysis. J. Colloid Interface Sci. **679**, 481–489 (2025). <https://doi.org/10.1016/j.jcis.2024.10.128>
14. L. Ye, Z. Chen, X. Xu, F. Ma, K. Fan et al., Ultrafast room-temperature synthesis of phosphate-intercalated NiFe layered double hydroxides for high-performance alkaline seawater oxidation. Inorg. Chem. **63**(43), 20859–20869 (2024). <https://doi.org/10.1021/acs.inorgchem.4c03660>
15. X. Wang, H. Hu, J. Song, J. Ma, H. Du et al., Surface anticorrosion engineering by polyphosphate oxyanions for durable seawater oxidation. Adv. Energy Mater. **15**(4), 2402883 (2025). <https://doi.org/10.1002/aenm.202402883>
16. T. Kanwar, P. Arun, R. Silviya, A. Bhide, S. Gupta et al., Optimized trimetallic CoNiFe phospho-boride electrocatalyst for overall seawater electrolysis. J. Power Sources **633**, 236427 (2025). <https://doi.org/10.1016/j.jpowsour.2025.236427>
17. H. Chen, R. Cheng, M. Jiang, X. Shao, X. Zhang et al., Silver induced in-phase electronic interaction and chloride ion repelling for efficient electrocatalytic oxygen evolution in seawater electrolysis. ACS Appl. Energy Mater. **8**(6), 3416–3424 (2025). <https://doi.org/10.1021/acsaem.4c02942>
18. G. Wang, W. Tang, Y. Chen, P. Ji, M. Lu et al., The Ni heteroatom-induced electronic structure tailoring of ultrastable Fe_3_N@NCPs nanosheets electrocatalyst for boosting alkaline seawater electrolysis. Adv. Funct. Mater. **34**(39), 2404470 (2024). <https://doi.org/10.1002/adfm.202404470>
19. M. Wei, J. Wang, X. Ma, Y. Cao, X. Yuan et al., Lattice Cl^–^ reconstruction in a ternary hydroxychloride pre-electrocatalyst for efficient saline water oxidation. Carbon Future **2**(3), 9200052 (2025). <https://doi.org/10.26599/cf.2025.9200052>
